# Towards the First Multiepitope Vaccine Candidate against *Neospora caninum* in Mouse Model: Immunoinformatic Standpoint

**DOI:** 10.1155/2022/2644667

**Published:** 2022-06-09

**Authors:** Morteza Shams, Bahman Maleki, Bahareh Kordi, Hamidreza Majidiani, Naser Nazari, Hamid Irannejad, Ali Asghari

**Affiliations:** ^1^Zoonotic Diseases Research Center, Ilam University of Medical Sciences, Ilam, Iran; ^2^Department of Parasitology, Faculty of Medical Sciences, Tarbiat Modares University, Tehran, Iran; ^3^Department of Basic Medical Sciences, Neyshabur University of Medical Sciences, Neyshabur, Iran; ^4^Department of Parasitology and Mycology, School of Medicine, Kermanshah University of Medical Sciences, Kermanshah, Iran; ^5^Department of Medicinal Chemistry, Faculty of Pharmacy, Mazandaran University of Medical Sciences, Sari, Iran; ^6^Pharmaceutical Sciences Research Center, Mazandaran University of Medical Sciences, Sari, Iran; ^7^Department of Medical Parasitology and Mycology, School of Medicine, Shiraz University of Medical Sciences, Shiraz, Iran

## Abstract

*Neospora caninum* is an economically significant parasite among livestock, particularly in dairy cattle herds, causing storm abortions. Vaccination seems necessary to limit the infection and its harsh consequences. This is the first steps towards developing a multiepitope vaccine candidate against *N. caninum* using in silico approaches. High-ranked mouse MHC-binding and shared linear B-cell epitopes from six proteins (SRS2, MIC3, MIC6, GRA1, IMP-1, and profilin) as well as IFN-*γ*-inducing epitopes (from SAG1) were predicted, screened, and connected together through appropriate linkers. Finally, RS-09 protein (TLR4 agonist) and histidine tag were added to N- and C-terminal of the vaccine sequence, yielding 486 residues in length. Physicochemical properties showed a stable (instability index: 27.23), highly soluble, antigenic (VaxiJen score: 0.9554), and nonallergenic candidate. Secondary structure of the multiepitope protein included 58.85% random coil, 20.99% extended strand, and 20.16% alpha helix. Also, the tertiary structure was predicted, and further analyses validated a stable interaction between the vaccine model and mouse TLR4 (binding score: -1261.6). Virtual simulation of immune profile demonstrated potently stimulated humoral (IgG+IgM) and cell-mediated (IFN-*γ*) responses upon multiepitope vaccine injection. Altogether, a potentially immunogenic vaccine candidate was developed using several *N. caninum* proteins, with the capability to elicit IFN-*γ* upsurge and other components of cellular immunity, and can be used in prophylactic purposes against neosporosis.

## 1. Introduction

The cyst-forming obligatory intracellular protist, *Neospora caninum* (*N. caninum*), is the causative agent of neosporosis [1] and a major cause of epidemic and/or endemic abortions among livestock, particularly in dairy cows [2, 3]. It is estimated that over US$1 billion are wasted annually in both dairy and beef cattle industries due to *N. caninum* infections [4]. The parasite circulates between wild/domestic canids, as definitive hosts, including dogs (*Canis familiaris*) [5], coyotes (*Canis latrans*) [6], dingoes (*Canis dingo*) [7], and gray wolves (*Canis lupus*) [8], and intermediate herbivorous hosts such as cattle and water buffalo (*Bubalus bubalis*) [9]. Based on a recent systematic review, the pooled prevalence of *N. caninum* infection among dogs was estimated to be 17.14% (95% confidence interval: 15.25% -1910%) worldwide [10]. Similar to the sibling coccidian parasite, *Toxoplasma gondii*, *N. caninum* oocysts shed *via* canid feces are sporulated under optimum bioclimatic conditions, being infectious for both canids and herbivores [11]. Exogenous (oocyst-derived) or endogenous (tissue cyst-derived) transplacental infections are the primary route of parasite circulation and propagation among cattle herds [12, 13]. Reproductive failure and fetal death are the direct principal economic consequences of neosporosis in cattle, along with indirect losses such as veterinary care [14] as well as replacing and rebreeding of culled animals [15]. Up to 95% of the progenies of seropositive cattle are seemingly healthy without clinical abnormalities, but they are carriers actually that will infect their progeny later in their life [16, 17].

Economically, treating seropositive animals may not seem rational, due to the lack of effective and safe drugs as well as long-time treatment regimen, which is unfavorable regarding drug residues in cattle productions [15, 18]. Preventive measures such as vaccination appear to be more advantageous and economic sense, without the risk of drug residues in food animals [19, 20]. An ideal vaccination platform against *N. caninum* should fulfill the following issues: (i) prevention of tissue cyst formation in food animals to break off the transmission through carnivorism, (ii) decreased or abolished oocyst shedding in final hosts, and (iii) inhibiting tachyzoite propagation in pregnant cattle to prevent congenital transmission [21]. This aim is achieved *via* a vaccine candidate that stimulates protective cellular and antibody-dependent components at both mucosal and systemic levels [22]. Different vaccines have been investigated against the infection in cattle and mouse models, such as the application of naturally less virulent isolates and attenuated strains [23]; the latter has shown promising efficacies in both cattle and mice, despite safety concern and production costs [21]. Subunit peptide-based or DNA vaccines have been more focused during last decades, due to their explicit benefits in reduced production, processing, and storage costs along with higher shelf-life and stability [24]. In this sense, most studies have been done using those molecular targets involved in adhesion/invasion processes, encompassing surface antigens, microneme (MIC) and rhoptry (ROP) proteins, dense granular (GRA) components, as well as various molecules in parasitophorous vacuole membrane (PVM) [25].

Conventional vaccine development is a time-consuming and costly practice, involving laborious experimental work [26]. *In silico* pipelines are emerging computer-based practices for high-throughput structure-based vaccinology purposes *via* engineering multiepitope constructs and optimizing their immunogenic and biochemical performances [27]. Previously, several *N. caninum* antigens were validated as possible vaccine targets, including SAG1, SAG1-related sequence 2 (SRS2) [28], MIC3 [29], MIC6 [30], GRA1 [31], Immune-Mapped Protein 1 (IMP-1) [32], and profilin [33]. Nevertheless, the mouse-specific immunogenic epitopes of these vaccine candidates have not been determined yet. The present study was aimed towards engineering the first multiepitope vaccine candidate using the epitopes derived from these proteins by applying comprehensive immunoinformatic approaches.

## 2. Methods

### 2.1. Retrieval of *N.* caninum Protein Sequences

The protein sequences of *N. caninum* SAG1 (accession number: Q9UB12), SRS2 (accession number: Q58L77), GRA1 (accession number: P90661), MIC3 (accession number: F0VAA2), MIC6 (accession number: A0A0M4B3R2), IMP-1 (accession number: J9PWX7), and profilin (accession number: D6VPE7) were retrieved *via* the UniProt Knowledgebase (UniProtKB), as a freely-accessible resource for protein sequences and biological functions (https://www.uniprot.org/) [34]. Of note, the protein sequence of SAG1 was only used for the prediction of IFN-*γ*-inducing epitopes.

### 2.2. Multistep Prediction and Screening of Continuous B-Cell Epitopes

For linear B-cell epitope prediction, three web servers were employed, including BCPREDS (http://ailab.ist.psu.edu/bcpred/predict.html), ABCpred (http://crdd.osdd.net/raghava/abcpred/), and SVMTriP (http://sysbio.unl.edu/SVMTriP/index.php), all being trained to differentiate B-cell from non-B-cell epitopes through machine learning- (ML-) based methods. The BCPREDS server combines support vector machine (SVM) with subsequent kernel (SSK) with 74.75% prediction accuracy [35], while SVMTriP server employs SVM and Tri-peptide similarity and propensity scores [36]. Also, the ABCpred server exploits artificial neural networks (ANNs) for fixed-length epitope prediction, showing 65.93% accuracy [37]. In this study, a fixed-length (14 amino acids) prediction was applied to all servers, with a prediction threshold of 75% and 0.80% for BCPREDS and ABCpred online servers, respectively. Upon selection of shared epitopes predicted by at least two web servers, they were further subjected to screening in terms of antigenicity, allergenicity, and water solubility, using VaxiJen v2.0 (http://www.ddg-pharmfac.net/vaxijen/VaxiJen/VaxiJen.html), AlleregnFP v1.0 (https://ddg-pharmfac.net/AllergenFP/), and PepCalc (https://pepcalc.com/) online tools, respectively. Finally, one common, highly antigenic, and nonallergenic linear B-cell epitope having good water solubility was chosen from each protein to be included in the vaccine model assemblage.

### 2.3. Prediction and Screening of Mouse Major Histocompatibility Complex- (MHC-) Binding Epitopes

For the prediction of 12-mer MHC-I and 15-mer MHC-II epitopes, the Immune Epitope Database (IEDB) MHC-I (http://tools.iedb.org/mhci/) and MHC-II (http://tools.iedb.org/mhcii/) tools were used. These tools utilize combined prediction methods, such as ANN and quantitative affinity matrix (QAM), applying the more appropriate for each MHC allele. The final output yields a percentile rank, having inverse correlation with epitope affinity, so that lower percentile ranks are associated with higher confidence for epitope affinity [38]. Epitope prediction for mouse MHC-I alleles (H2-Db, H2-Dd, H2-Kb, H2-Kd, H2-Kk, H2-Ld, H2-Qa1, and H2-Qa2) and MHC-II alleles (H2-IAb, H2-IAd, and H2-IEd) was done based on IEDB recommended 2020.04 (NetMHCpan EL 4.0) and IEDB recommended 2.22 methods, respectively. In the following, top high-ranked epitopes were screened regarding antigenicity, allergenicity, and toxicity using VaxiJen v2.0, AllergenFP v1.0, and ToxinPred (http://crdd.osdd.net/raghava/toxinpred) web servers, respectively. Subsequently, only one epitope with high antigenic index and without allergenicity and toxicity was selected from each examined protein for vaccine construction.

### 2.4. Prediction and Screening of IFN-*γ*-Inducing Epitopes from *N. caninum* SAG1

To predict those epitopes capable to induce IFN-*γ* cytokine epitopes in *N. caninum* SAG1 protein sequence, as a major arm of Th1 response, IFN epitope server was used with a hybrid (motif and SVM) approach. Twelve peptides with higher scores were selected and subsequently screened in terms of antigenicity, allergenicity, and toxicity through VaxiJen v2.0, AllergenFP v1.0, and ToxinPred servers, respectively.

### 2.5. Engineering and Assemblage of the Multimeric Vaccine Candidate Sequence

After a computer-based multistep prediction and screening of specific epitopes, the putative multiepitope vaccine sequence was constructed using two MHC-I, one MHC-II and one linear B-cell epitopes from six examined *N. caninum* proteins (SRS2, GRA1, MIC3, MIC6, IMP-1, and profilin) along with three potential IFN-*γ*-inducing epitopes derived from *N. caninum* SAG1. The immunogenicity of the vaccine model was enhanced by the addition of RS-09 peptide adjuvant (APPHALS), as a synthetic toll-like receptor 4 (TLR4) agonist [39]. The adjuvant sequence (N-terminus) was linked to the first MHC-I epitope through an “EAAAK” spacer. Moreover, MHC-I epitopes were joined together using “AAY” linker, while MHC-II, linear B-cell, and IFN-*γ*-inducing epitopes were fused with “GPGPG” linker. Notably, a 6 × histidine (His-tag) sequence (CATCACCATCACCATCAC) was added to the C-terminus of the vaccine model for future protein purification.

### 2.6. Physicochemical Characteristics of the Final Vaccine Construct

In order to predict the basic physicochemical features of the engineered vaccine model, comprising molecular weight (MW), number of negatively and positively charged residues, aliphatic and instability indices, isoelectric point (pI), and half-life and grand average of hydropathicity (GRAVY), ExPASy ProtParam server was used, available at https://web.expasy.org/protparam/. Stability of a given protein in test tube is determined *via* instability index, while aliphatic side chain mass important regarding protein thermotolerance is appointed to aliphatic index. The pI concept devotes to a pH value at which net charge turns zero. Also, GRAVY is assigned to the average hydropathicity values of the protein residues [40].

### 2.7. Multiserver Prediction of Antigenicity, Allergenicity, and Solubility Profiles

Antigenicity is a primary characteristic of a vaccine candidate, so we employed two online tools for this purpose: VaxiJen v2.0 (http://www.ddg-pharmfac.net/vaxijen/VaxiJen/VaxiJen.html) and ANTIGENpro of SCRATCH Protein Predictor Suite (http://scratch.proteomics.ics.uci.edu/). The alignment-free VaxiJen v.20 server works on the basis of protein sequence transformation into uniform vectors of major amino acid properties using auto cross covariance (ACC) [41]. Also, ANTIGENpro is a pathogen-independent, alignment-free predictor of antigenicity using a two-stage architecture and five ML algorithms, trained by reactivity information obtained from protein microarray analyses for five pathogens [42]. Lack of allergenic traits is another crucial feature of a vaccine candidate, which was assessed using three web servers: AllergenFP v1.0 (https://ddg-pharmfac.net/AllergenFP/), AllerTOP v2.0 (https://www.ddg-pharmfac.net/AllerTOP/), and AlgPred (http://crdd.osdd.net/raghava/algpred/). The AllergenFP v1.0 “differentiates allergens from antigens utilizing a four-step, alignment-independent, and descriptor-based fingerprint method with 88.9% accuracy using Mathews correlation coefficient of 0.759” [43]. Another server developed by Dimitrov *et al.*, AllerTOP v2.0, employs and combines several ML techniques for sorting allergens, such as*k*-nearest neighbors, amino acid E-descriptors, as well as auto and cross-variance transformation [44]. AlgPred server presents several prediction approaches such as mapping IgE epitopes, MEME (Multiple Em for Motif Elicitation)/MAST (Motif Alignment and Search Tool) allergen motifs, Blast search on allergen representative peptides (ARPs), and hybrid method [45]. Here, we used two first approaches (IgE and MEME/MAST) to render the protein as a nonallergen. Protein solubility prediction was done *via* two servers: Protein-Sol (https://protein-sol.manchester.ac.uk/) and SOL-pro (http://scratch.proteomics.ics.uci.edu/). Regarding Protein-Sol, a threshold score of 0.45 is assigned to the population average of the experimental dataset, so higher values indicate to highly soluble proteins [46]. Also, SOL-pro exerts a two-stage SVM method to estimate the solubility upon overexpression in *Escherichia coli* (*E. coli*) [47].

### 2.8. Extrapolation of the Secondary Structure

For this aim, two web servers were used, including Garnier–Osguthorpe–Robson (GOR IV) (https://npsa-prabi.ibcp.fr/cgi-bin/npsa_automat.pl?page=/NPSA/npsa_gor4.html) and NetSurfP-2.0 (https://services.healthtech.dtu.dk/service.php?NetSurfP-2.0). GOR server provides sequence- and graphical-based prediction, including percentages of alpha helix, 3_10_ helix, Pi helix, beta bridge, extended strand, beta turn, bend region, random coil, ambiguous, and other states [48]. The NetSurfP-2.0 server predicts surface accessibility, secondary structure, disordered regions, and phi and psi angles. A single model, using a combination of convolutional and bidirectional long-short term memory neural networks, predicts all structural features together [49].

### 2.9. Prediction, Refinement, and Validation of the Three-Dimensional (3D) Vaccine Model

An Iterative Treading ASSEmbly Refinement (I-TASSER) modality was employed for a fine tuned, automated homology modelling of the engineered vaccine candidate (https://zhanglab.dcmb.med.umich.edu/I-TASSER/) [50–52]. In the following, selected 3D model was further subjected to rehashing process through DeepRefiner server, available at http://watson.cse.eng.auburn.edu/DeepRefiner/. The refinement process was performed using “Residual Neural Networks (ResNet)” as deep learning model and “adventurous” refining mode using noncumulative restraints. A higher predicted global quality score and lower values of Rosetta energy, GOAP, OPSUS-PSP, DFIRE, MolProbity, and RWPlus render a high quality model [53]. Furthermore, the refined model was validated using three web server, including ERRAT and PROCHECK tools of SAVES 6.0 server (https://saves.mbi.ucla.edu/) and Prosa-Web (https://prosa.services.came.sbg.ac.at/prosa.php). “The ERRAT tool of SAVES v6.0 server explores the statistics of nonbonded atom to atom interactions and depicts the error function value *versus* position of a 9-residue sliding window, estimated by a comparison with statistics from high resolution crystallography structures” [54]. Prosa-Web estimates the total quality of the submitted 3D model regarding all known protein structures using a Z-score [55]. Moreover, “PROCHECK visualizes allowed (psi, *ψ*) and disallowed (phi, *φ*) dihedral angles of an amino acid residue *via* creating Ramachandran plots, based on van der Waal radius of the side chains” [56].

### 2.10. Prediction of Conformational B-Cell Epitopes

Nonlinear B-cell epitopes were predicted in the multiepitope vaccine sequence using one of the best online tools, ElliPro of the IEDB server, available at http://tools.iedb.org/ellipro/. The server has a significant AUC of 0.732, and default settings of 0.5 min score and 6 Å max-distance were applied. The output is shaped in a three-step process: calculation of protein shape, residual protrusion index (PI), and neighbor residue clustering. Of note, those residues with higher scores may be associated with enhanced solvent accessibility [57–59].

### 2.11. Vaccine Protein Disulfide Engineering

The probability of cysteine bond formation in the final chimeric vaccine sequence, disulfide engineering tool of DbD2 server (http://cptweb.cpt.wayne.edu/DbD2/index.php) was employed. A cysteine mutation is applied to all residues localized to the highly mobile area of the sequence. Residues were screened regarding the following parameters: <2.5 B-factor energy value (Kcal/mol) and *-87* to *+*97 chi^3^ value. Disulfide bonds, if present, would improve the protein geometric conformation and total stability [60].

### 2.12. Interaction between the Vaccine Model and Mouse TLR4

The 3D structure (pdb file) of mouse TLR4 (accession number: 3VQ2) was retrieved from the PDB database of Research Collaboratory for Structural Bioinformatics (RCSB), being available at https://www.rcsb.org. In the following, a protein-protein docking was done using ClusPro 2.0 web server with default settings, to estimate the binding affinity between the refined vaccine structure and mouse TLR4 [61]. The server output provides top-rank clusters, among which the most appropriate docking pose was selected for visualization.

### 2.13. Reverse Translation, Codon Adaptation, and In Silico Cloning

Efficiently higher yields of the protein produced in *E. coli* expression system are crucial for subunit vaccine production. For this purpose, reverse translation and codon optimization were done using reverse translate tool of sequence manipulation suite (https://www.bioinformatics.org/sms2/rev_trans.html) and JCat server (http://www.jcat.de/), respectively. JCat shows GC content and codon adaptation index (CAI) of a given DNA sequence, being important for a high-throughput expression in the respective host. Here, we optimized the codons for enhanced protein expression in *E. coli* K12 strain. Finally, propercutting sites of *Eco53KI* and *EcoRV* restriction enzymes were added to the 5′ and 3′-OH of the codon adapted vaccine sequence, respectively.

### 2.14. Immune Simulation

The immune responses provoked by the finally approved, multimeric *N. caninum* vaccine model were predicted *in silico*, using C-ImmSim web server, available at http://150.146.2.1/C-IMMSIM/index.php.This virtual simulation process was accomplished using default parameters with random seed 12345, simulation volume 10, and simulation steps 100. A PSSM for ML methods are the basis for the predictions in this server. The output indicates to three stimulated regions including bone marrow, thymus, and lymph node [62].

## 3. Results

### 3.1. Linear B-Cell Epitope Prediction and Screening

Following a cross-validating approach to explore shared continuous B-cell epitope prediction and subsequent screening, one highly antigenic, nonallergenic, and well-soluble epitope was selected from each protein (VaxiJen scores in parenthesis): “DDAAGNPVDSD” from GRA1 (1.4553), “SEGQPCRNRQLHT” from MIC3 (1.2169), “GESGEGEE” from MIC6 (3.0951), “GPDGKAFPDDY” from SRS2 (1.6063), “MKYEQKGGKTE” from IMP-1 (1.5936), and “SKLYKEDHEEDT” from profilin (0.9530) (Supplementary File 1).

### 3.2. Mouse MHC-Binding and IFN-*γ* Epitope Prediction and Screening

Mouse MHC-I binding epitopes (12-mer) were predicted using IEDB server and screened regarding antigenicity, allergenicity, and toxicity. From each protein, two epitopes were finally qualified to be included in the final vaccine construct, as follows (VaxiJen scores in parenthesis): “AVPVVGALTSYL” (0.7984) and “TEQHEGDIGYGV” (1.2574) from GRA1; “AQLENSQHVEGV” (1.1212) and “NEKCGSNGSCIV” (1.5425) from MIC3; “YTPVNGRGGLTC” (0.7606) and “LWLQNDPRFFVL” (1.5742) from MIC6; “GHPDDKQVTCVV” (1.7670) and “VAHCAYSSNVRL” (1.5329) from SRS2; “EEEKAGKILVSF” (1.1575) and “LPRDRPVDLSVF” (0.9249) from IMP-1; and “VDDGSAPNGVWI” (1.1841) and “SRTSALAFAEYL” (0.8596) from profilin (Supplementary File 2).

Furthermore, one antigenic, nonallergenic, and nontoxic MHC-II binding epitope (15-mer) was selected from each protein sequence, as follows (VaxiJen scores in parenthesis): “KKRVKTAVGIAALVA” from GRA1 (0.8230); “ALSPSFLASGISSEV” from MIC3 (1.1256); “GKKEESKGSAAAIAG” (1.5561) from MIC6; “AHCAYSSNVRLRPIT” (1.4654) from SRS2; “DLSVFSHVAVVPADK” (0.5379) from IMP-1; and “GVWIGGQKYKVVRPE” (0.9887) from profilin (Supplementary File 3).

Top five IFN-*γ*-inducing epitopes with potential antigenicity and without allergenicity and toxicity were selected from the *N. caninum* SAG1 protein sequence, as follows (VaxiJen scores in parenthesis): “PRAVRRAVSVGVFAA” (0.5884), “EAERASAGIKSSAEN” (0.9542), and “ASAGIKSSAENVGRV” (0.6057) (Supplementary File 4).

### 3.3. Construction of the Vaccine Model and Physicochemical Assessment

**Figure 1:**
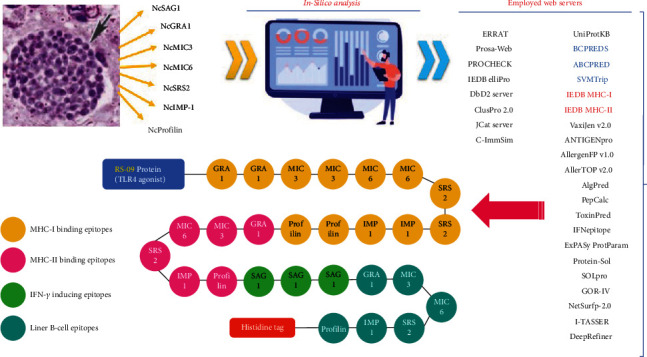
Schematic representation of the multiepitope vaccine design steps [63]. Blue and red web servers were used in B- and T-cell epitope predictions, respectively. Also, TLR4 agonist and histidine tag are attached to the N-terminal and C-terminal of the vaccine sequence, respectively.

Our designed multiepitope vaccine construct included 486 residues with five domains comprising T-cell and IFN-*γ*-inducing and B-cell epitopes, followed by a TLR4 agonist (RS-09 peptide) at N-termini and a so-called His-tag at C-termini (Figure 1). The antigenicity of the crude sequence was 0.9502, being optimized to 0.9554 after addition of adjuvant and His-tag sequences. According to ExPASy ProtParam server, a MW of 48484.93 dalton, with 47 and 39 negatively (Asp + Glu)- and positively (Arg+Lys)-charged residues were estimated for the putative protein. Moreover, the speculated pI was 5.68, with a 27.23 instability index, 63.37 aliphatic index, and -0.244 GRAVY score. The estimated half-life of the protein was 4.4 hours (mammalian reticulocytes, *in vitro*), >20 hours (yeast, *in vivo*), and >10 hours (*E. coli*, *in vivo*).

### 3.4. Antigenicity, Allergenicity, and Solubility of the Multiepitope Vaccine Model

Based on AllergenFP v1.0 and AllerTOP v2.0 servers, the multiepitope protein was nonallergen in nature; also, AlgPred server demonstrated that there is no specific IgE epitopes and MEME/MAST motifs in the protein sequence, rendering it as a nonallergen molecule. The protein was demonstrated as a potent antigenic molecule based on VaxiJen scores of 0.9502 (crude sequence) and 0.9554 (with adjuvant and H6-tag). In addition, ANTIGENpro showed the probability of antigenicity of the protein as 0.905345 (highly antigen). Finally, designed protein sequence was highly soluble, according to 0.927147 and 0.519 scores obtained by SOLpro and Protein-Sol servers, respectively.

### 3.5. Secondary Structure Prediction

**Figure 2:**
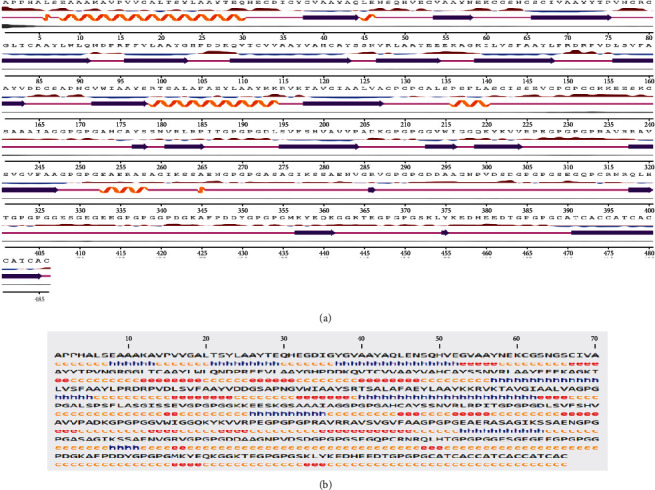
The graphical output of the secondary structure analysis provided by (a) NetSurfP-2.0 and (b) GOR IV web servers.

Secondary structure of the putative protein was determined using GOR IV server, showing 286 (58.85%) random coil, 102 (20.99%) extended strand, and 98 (20.16%) alpha helix. This finding was, again, confirmed using NetSurfP-2.0 server. This server, also, revealed that most of the residues are exposed regarding surface accessibility and there were no disordered regions in the main sequence residues, except for adjuvant area. The graphical illustration of the secondary structure analysis is provided in Figure 2.

### 3.6. 3D Model Prediction, Refinement, and Validations

**Figure 3:**
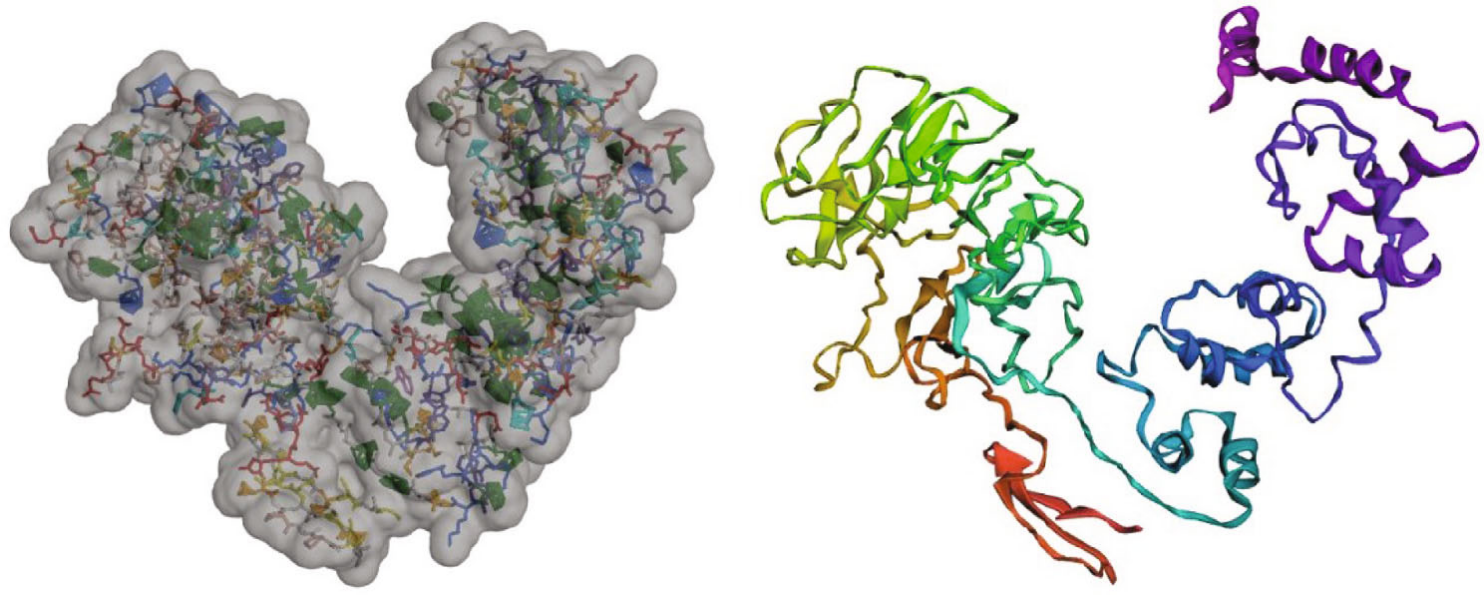
The 3D model of the multiepitope vaccine construct, predicted by I-TASSER server.

**Figure 4:**
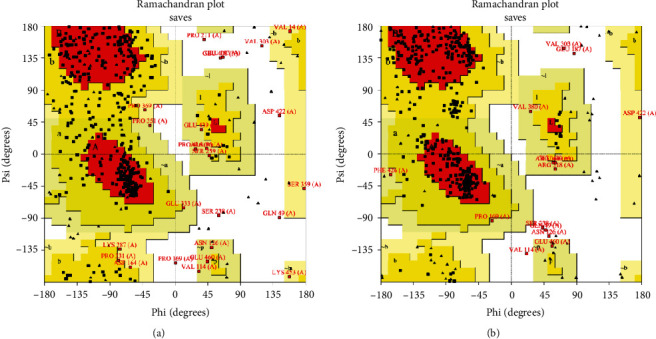
The outputs of PROCHECK server for Ramachandran plot analysis. (a) In crude model, 61.5%, 33.4%, 3.4%, and 1.7% of the residues were in most favored, additional allowed, generously allowed, and disallowed regions, respectively. (b) In the refined model, 75.0%, 21.3%, 2.0%, and 1.7% of residues were located at most favored, additional allowed, generously allowed, and disallowed regions, respectively.

Homology modelling of the multimeric protein was done using LOMETS metaserver threading approach of I-TASSER server, yielding top 10 threading templates and 5 most suitable 3D models. The C-score is usually used to identify the best-fit 3D model, typically ranging from -5 to 2; higher C-score indicates to higher confidence of prediction. In present modelling study, the C-scores ranged from -1.49 to -3.92, among which model number one was chosen as the best 3D model for our chimeric vaccine, with a C-score of -1.49, estimated TM-score of 0.53 ± 0.15, and estimated RMSD of 10.7 ± 4.6 Å (Figure 3). “Imbalances related to RMSD values are dissolved using TM-score *via* analyzing for similarities between two protein structures. In this sense, TM-scores above 0.5 mean accurate topology, while less than 0.17 values show nonspecific similarity.” In the next step, 3D model number 1 was further subjected to refinement process and subsequent validations. Based on DeepRefiner server output, among five refined models provided, mode number 5 was selected as the finally refined vaccine model, with predicted global quality score of 0.040, Rosetta energy score of -262.272, MolProbity score of 1.996, GOAP score of -23121.070, OPSUS-PSP score of -3579.560, DFIRE score of -518.484, and RWPlus score of -65264.948. The improvement in the vaccine sequence was prominent, as evidenced by 78.478 (crude model) vs 86.486 (refined model) quality factor obtained by ERRAT tool, as well as -2.57 (crude model) vs -3.64 (refined model) Z-scores provided by Prosa-Web server. Moreover, the PROCHECK server results showed an improved model including 75.0%, 21.3%, 2.0%, and 1.7% residues in the most favored, additional allowed, generously allowed, and disallowed regions, respectively (Figure 4).

### 3.7. Prediction of Conformational B-Cell Epitopes

**Figure 5:**
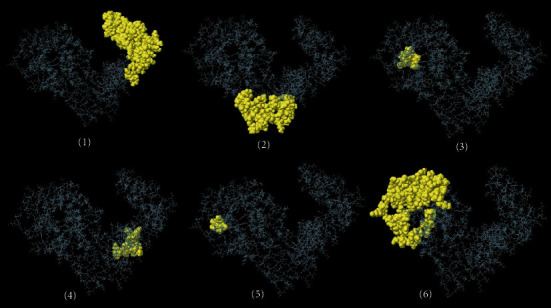
Conformational B-cell epitopes predicted by ElliPro online tool. The number of residues and score for each epitope are as follows: (1) 96 residues, score: 0.757; (2) 54 residues, score: 0.723; (3) 9 residues, score: 0.658; (4) 12 residues, score: 0.652; (5) 5 residues, score: 0.644; and (6) 101 residues, score: 0.631.

Based on ElliPro server output, six potential conformational B-cell epitopes were present in the vaccine sequence, as follows: (i) 96 residues, score: 0.757; (ii) 54 residues, scores: 0.723; (iii) 9 residues, score: 0.658; (iv) 12 residues, score: 0.652; (v) 5 residues, score: 0.644; and (vi) 101 residues, score: 0.631 (Figure 5).

### 3.8. Vaccine Protein Disulfide Engineering

As estimated by DbD2 server, 67 pairs of amino acid residues were potentially examined regarding disulfide bond formation in the finally refined vaccine construct. Only those residues having -87 to +97 chi^3^ value and a <2.5 B-factor energy (Kcal/mol) satisfied the band establishment. On this basis, 8 residue pairs were qualified for disulfide band engineering, including GLY 33–VAL 39, GLN 44–ALA 56, TYR 72–VAL 76, GLU 135–TPR 173, PRO 311–VAL 324, ALA 334–ALA 337, GLU 457–CYS 478, and CYS 475–CYS 481.

### 3.9. Protein-Protein Docking Analysis with Mouse TLR4

**Figure 6:**
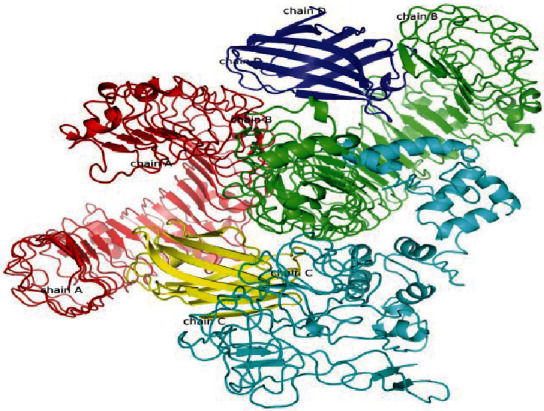
Docked conformation of the chimeric vaccine and mouse TLR4. The designed vaccine is in cyan blue, while mouse TLR4 is in red (chain A), green (chain B), yellow (chain C), and blue (chain D). The chimeric protein showed interactions with chains B and C.

**Figure 7:**
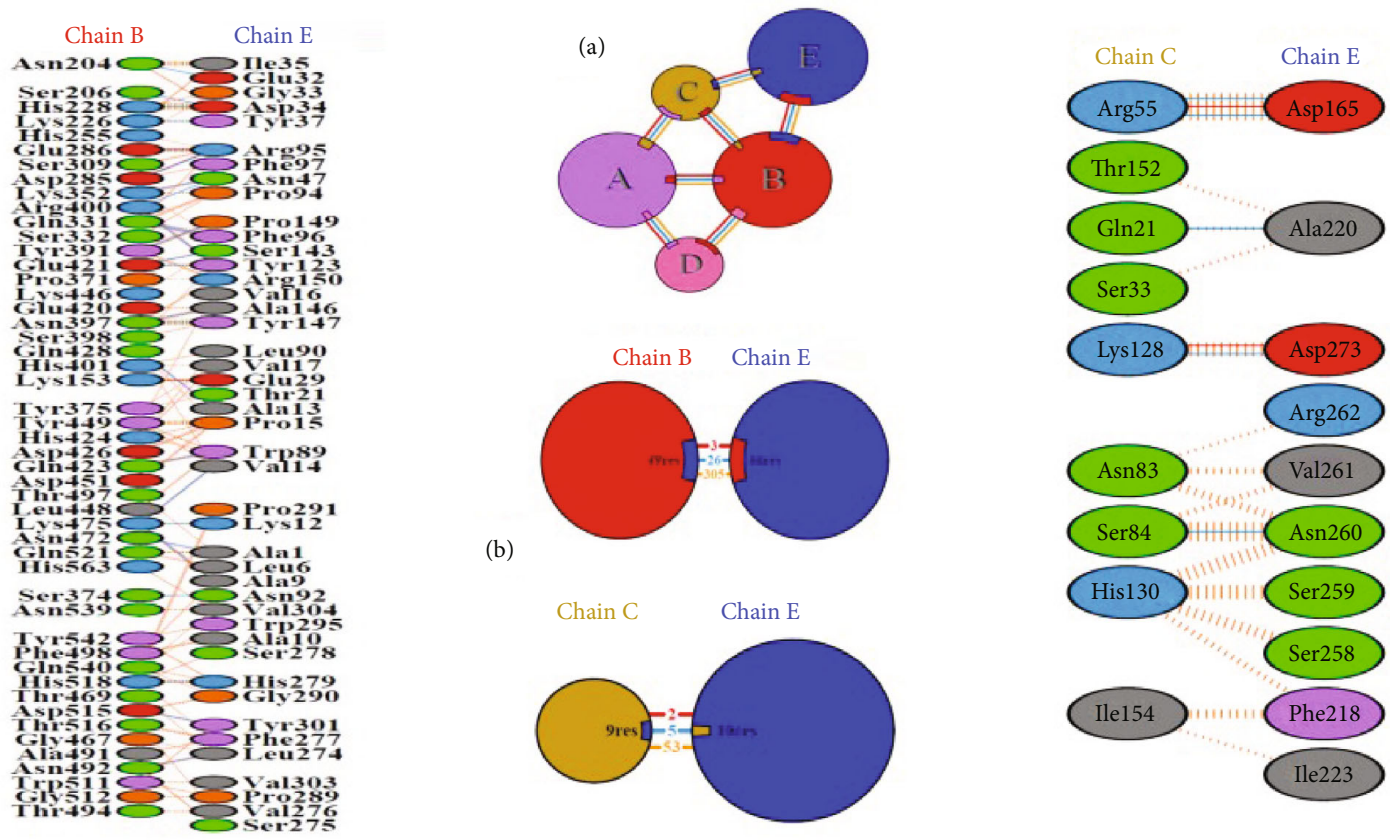
Schematic representation of the molecular interactions between the chimeric vaccine and mouse TLR4, where E blue sphere is the chimeric vaccine and other four spheres belong to the receptor. (a) Interacting chains are linked by colored lines, each representing different type of interaction. Red denotes for salt bridges, blue for hydrogen bonds, and orange for nonbonded contacts. The size of each circle is proportional to the surface area of the corresponding protein chain. The extent of the interface region contacting the other chain is represented by a colored wedge whose color corresponds to the color of the other contacting chain and whose size signifies the interface surface area; (b) Blue line denotes hydrogen bond, red denotes salt bridge, and orange striped lines denote nonbonded contacts. In cases which the nonbonded contacts are plentiful, the width of the striped line increases depending on the number of the nonbonded contacts.

The piper-based ClusPro 2.0 web server provided a number of docking poses between the mouse TLR4 molecule (PDB code: 3VQ2) as receptor and our designed multiepitope vaccine construct. The first ranked, highly populated docking cluster with the highest binding score (-1261.6) was chosen for further visualization of the molecular interactions and binding conformation. As illustrated in Figure 6, the molecular interaction between two docked molecules was observed in chains B and C of the TLR4 with vaccine construct. Moreover, the molecular and amino acid interactions between the docked vaccine and the receptor are precisely illustrated in Figure 7.

### 3.10. Codon Optimization and In Silico Cloning

Reverse translation of the protein sequence into the DNA sequence was done using the reverse translate tool of the sequence manipulation suite. In the following, the sequence was subjected to the JCat server for codon optimization and enhanced expression level in *E. coli* K12 strain. The CAI value and GC% of the initially submitted sequence were 0.53 and 67.76, respectively, whereas these were improved in the codon optimized sequence as 1.0 and 55.06, correspondingly. An optimum score of 30%-60% for GC content and 08-1 for CAI value may lead to enhanced protein expression in a given host organism. These obtained results suggest that the expression of enhanced DNA sequence of the vaccine is maximum in the selected host. Since the cutting sites of the *Eco53KI* and *EcoRV* restriction enzymes were not present in the sequence, cutting sequences as well as Shine-Dalgarno (AGGAGG) and start-stop codon sequences were embedded for final *in silico* cloning procedure.

### 3.11. Simulation of Immune Profile

**Figure 8:**
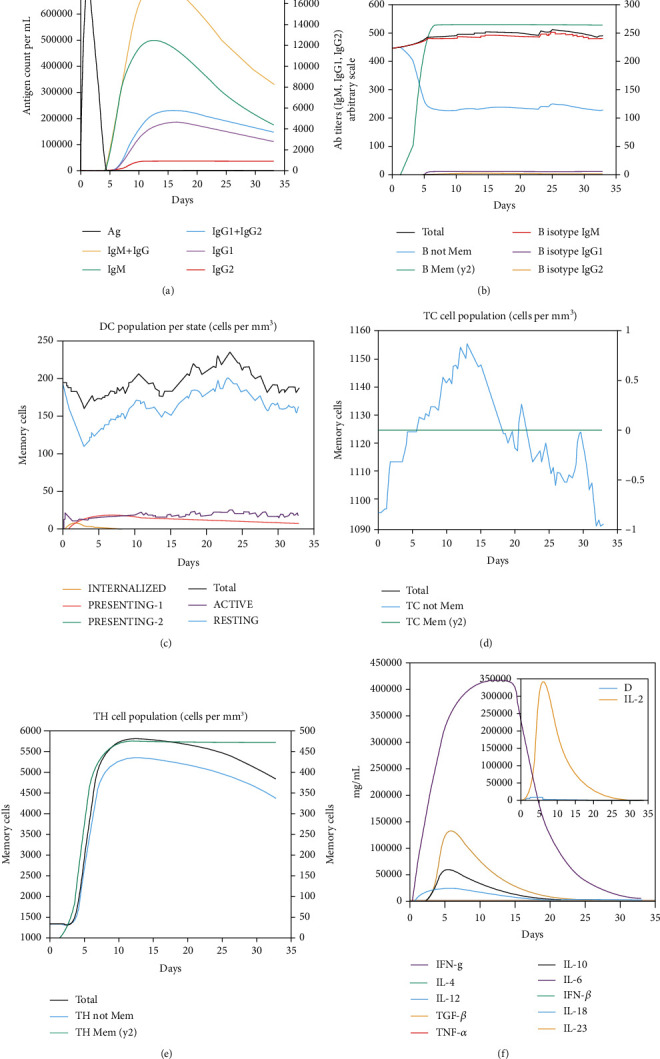
Virtual immune simulation with the designed subunit vaccine. (a) Immunoglobulin production in response to antigen; (b) B-cell populations (cells per mm^3^); (c) DC cell population per state (cell per mm^3^); (d) T cytotoxic cell population (cells per mm^3^); (e) T helper cell population (cells per mm^3^); and (f) Level of cytokines induced by the chimeric vaccine.

The humoral and cell-mediated immune responses were considerably stimulated upon multiepitope vaccine injection, as evidenced in C-ImmSim server outputs. Noticeably, B memory cells increased rapidly to over 250 cell/mm^3^ 5 days after vaccine injection and remained steadily. Also, an initially high isotype IgM B-cells were demonstrated to be present over 30 days. Antibody response graph showed a remarkably high IgM and IgM+IgG titers raised against the antigenic vaccine. A low number of dendritic cells (DCs), about 20 cells/mm^3^, were active and always presenting the antigen to the immune cells for over a month upon vaccine injection. Of note, helper T memory cells increased rapidly during 5 days to over 450 cell/mm^3^ and thereafter remained. Nonmemory cytotoxic T-cells were also prominent between 6 and 17 days upon antigen exposure. A very strong IFN-*γ* response (over 400000 ng/ml) was evident among other cytokines, increased rapidly from days 0 until 16 upon exposure (Figure 8).

## 4. Discussion

First insights into the immunobiology of the apicomplexan parasite, *N. caninum*, in cattle and dogs were revealed during 1999 to 2003 [64], leading to the emergence of the initial vaccination approaches in the mouse model [65] and in cattle as target species [66]. In parallel with deciphering the parasite biology and identification of parasitic antigens, more studies on *N. caninum* vaccination were performed during the last decade, using novel antigens and different immunization platforms. Having no live component, subunit vaccines entail no risk of disease; hence, they are highly interested for a safe vaccination approach, usually accompanied by an adjuvant as a potent immune enhancer [27]. Innovative technology-based methods such as reverse vaccinology and immunomics have facilitated the accurate screening and selection of potential antigenic targets and antigenic epitopes among multiple proteins [27, 67, 68]. Among a plethora of employed vaccine candidates in *N. caninum*, six proteins (GRA1, MIC3, MIC6, SRS2, profilin, and IMP-1) were selected to further explore epitopic regions and assemble a novel multimeric vaccine candidate.

*Neospora caninum* SAG1 and SRS2 are principal immunodominant surface antigens in tachyzoites, which mediate initial low-affinity, reversible adhesion to the host cell prior to invasion [28]. A micronemal component, MIC3, is expressed on the parasite surface and enhances cellular attachment *via* four adhesive epidermal growth factor- (EGF-) like domains [29]. Another microneme-associated protein having EGF domains is MIC6 which physically interacts with MIC1 and MIC4, forming a stable complex [30]. Upon parasite entry, a parasitophorous vacuole is formed, in which several GRAs including GRA1 are secreted with possibly similar functions as is in *T. gondii* [31]. Profilin is highly homologous to its counterpart in *Toxoplasma*, being localized at the tachyzoite apical end with regulatory activities on actin polymerization. It is an essential protein for invasion, leading to the production of Th1-type cytokines (IL-2 and IFN-*γ*) *via* interaction with TLR11 and TLR12 [33]. Finally, IMP-1 gene is highly conserved among apicomplexan parasites, being newly discovered in *Eimeria maxima* in the last decade, and its protein is destined to localize on the tachyzoite surface [32]. Since mouse models are more accessible and affordable than cattle for immunity studies against neosporosis [69], we premised our multiepitope vaccine construct on mouse MHC-I and MHC-II binding epitopes, screened by antigenicity, allergenicity, and toxicity. Additionally, a wide array of stringent immunoinformatic-based filters from different online tools were applied to find shared B-cell epitopes, filtered by antigenicity, allergenicity, and water solubility screening. During early *N. caninum* infection, CD_4_^+^ Th1 polarization is a predominant response, leading to IFN-*γ* upsurge as a protective immune response [69]. Accordingly, IFN-*γ*-inducing epitopes of *N. caninum* SAG1 protein were, also, predicted and screened regarding antigenicity, allergenicity, and toxicity. Thereby, computers aided us to rationally develop a vaccine construct based on several high-ranked epitopes from six *N. caninum* proteins, which approved to be highly antigenic and without allergenicity.

The weak immunogenicity nature of the chimeric vaccines could be enhanced by addition of an adjuvant, which also prevents rapid degradation of the vaccine components and promotes the antigen delivery process to dendritic cells. Genetic adjuvants are one of the favorable components to be embedded in multiepitope vaccine constructs designed in silico [70]. Recently, TLR agonists have been developed and utilized to strongly activate antigen presenting cells (APCs) and the production of proinflammatory cytokines [71]. Since TLR4 is assumed to be relevant in protection against *N. caninum* [72, 73], a short-length synthetic TLR4 agonist peptide (RS-09: APPHALS) was added to the N-terminal of the final vaccine sequence. In fact, this peptide is a novel class of adjuvants that is considered as a mimotope to lipopolysaccharide substance of gram negative bacteria [39]. Linkers or spacers are short residues which separate domains within a given protein, to prevent unwanted molecular interactions and establishment of junctional epitopes or neoepitopes as well as to promote antigen presentation. In total, they actually improve the pharmacokinetic features and expression yield of the multiepitope vaccines [74]. In the present study, the adjuvant sequence was coupled with MHC-I binding epitopes using “EAAAK” linker [75]. Next, “AAY” linker as the proteasome cleavage site in mammalian cells joined MHC-I binding epitopes together [76], while “GPGPG” linker was employed to connect linear B-cell, MHC-II binding, and IFN-*γ*-inducing epitopes, which could beneficially enhance humoral responses [77]. Ultimately, the complete vaccine sequence was obtained by addition of a His-tag to the C-terminal, showing 486 residues in length.

Basic physicochemical characteristics of the vaccine construct are crucial for future experimental studies; hence, they were predicted using ProtParam web server. The multimeric vaccine construct designed in silico had a MW of 48.48 kDa, which could be employed as an indicator during SDS-PAGE electrophoresis and western blotting. Based on the estimated pI (5.68), the vaccine molecule was relatively acidic in nature, which could be directed towards ion-exchange chromatography and isoelectric focusing purposes. Moreover, it was presented as a moderately thermotolerant (aliphatic index: 63.37), stable (instability index: 27.23), and hydrophilic molecule (GRAVY score: -0.244). A proper vaccine candidate should not be allergenic in nature. In this sense, no IgE epitopes, MEME/MAST motifs, and allergenic traits were observed in the designed vaccine sequence. Moreover, potential antigenicity scores were obtained using VaxiJen server, either before (0.9502) and after (0.9554) addition of adjuvant and H6 sequences. The solubility of a protein depends on polar/nonpolar groups, amino acid composition, and molecular weight; our multiepitope vaccine protein was predicted to be soluble, according to SOLpro and Protein-Sol server output. Secondary structures of the vaccine protein constituted 58.85% random coil, 20.99% extended strand, and 20.16% alpha helix, most of which being exposed in terms of surface accessibility. In the next step, I-TASSER server created the best-fit 3D model of the chimeric vaccine (C-score: -1.49; TM-score: 0.53 ± 0.15, and RMSD value: 10.7 ± 4.6 Å), which further rehashed using GalaxyRefine web server. Afterwards, different web servers finally confirmed the quality of the refined model in comparison with the initially submitted model.

It is plausible that specific antibodies stimulated against *N. caninum* tachyzoites could beneficially inhibit the cellular invasion process [78]. Therefore, we predicted the conformational B-cell epitopes in the chimeric vaccine model using ElliPro online tool. The output represented six noncontinuous B-cell epitopes having 96, 54, 9, 12, 5, and 101 residues with 0.757, 0.723, 0.658, 0.652, 0.644, and 0.631 scores, respectively. It was shown that TLR4-deficient nonpregnant mice were highly susceptible and succumbed to the infection; hence, this innate receptor would play a critical role in appropriate sensing the parasite with subsequent induction of immune responses. Given this, a molecular docking was done between the multiepitope vaccine and mouse TLR4, using ClusPro 2.0 server, suggesting the presence of molecular interactions between the chimeric molecule and chains B and C of the receptor. Additionally, the simulated immune profile showed that DCs were actively presenting the antigen for over a month upon exposure and a good level of memory B-cells and helper T-cells was developed in response to the vaccine as well as a considerably high upsurge in IFN-*γ* response to eliminate the intracellular parasites.

Nowadays, bioinformatic-based procedures are more connected with the biological studies. In the field of vaccine design, immunoinformatics could substantially assist us for a rational in silico engineering of a vaccine model and prediction of its safety, stability, and efficacy, before any wet lab experiments. In the present study, a stringent, multistep process was applied to pick only those highly antigenic, nonallergenic epitopes with water solubility (B-cells) or without toxicity (MHC-binding and IFN-*γ* inducing). The secondary and 3D models of the final construct were predicted, refined, and validated and successfully docked with mouse TLR4, and the associated immune responses were simulated.

To our knowledge, the present study shows the first insights into the *in silico* multiepitope vaccine design against neosporosis in mouse model, using a set of online prediction web servers. The valuable findings of current study could be translated into the clinical settings, but it should be initially evaluated using *in vitro* assessment followed by *in vivo* challenge and protection experiments in standard laboratory models. Moreover, it is suggested to formulate the vaccine protein with other adjuvants and immunogenic epitopes derived from other potentially antigenic proteins.

It is, also, noteworthy that the current study met two major limitations, comprising (1) inaccessibility to accelerated, high-tech computer systems in order to run molecular dynamic simulation and predict the precise intermolecular interactions between the vaccine model and TLR4; and (2) focus on the limited number of proteins from *N. caninum* for multiepitope vaccine design.

## 5. Conclusion

In conclusion, a multiepitope vaccine construct was developed against *N. caninum* and assessed *in silico* using comprehensive immunoinformatic web servers. Based on our results, the vaccine candidate was shown to be highly antigenic and nonallergenic with good stability, solubility, and proper structural conformation, showing appropriate interaction with mouse TLR4 and stimulating adequate levels of humoral and cell-mediated immune responses to confine the infection, although wet laboratory experiments should confirm the actual efficacy of the engineered vaccine model.

## Data Availability

The data used to support the findings of this study are available from the corresponding author upon request.

## Supplementary Materials

Supplementary 1 Supplementary File 1. Predicted and screened common linear B-cell epitopes.

Supplementary 2 Supplementary File 2. Predicted and screened mouse MHC-I binding epitopes.

Supplementary 3 Supplementary File 3. Predicted and screened mouse MHC-II binding epitopes.

Supplementary 4 Supplementary File 4. Predicted and screened IFN-*γ*-inducing epitopes.
