# Supplementary material for: Towards the First Multiepitope Vaccine Candidate against Neospora caninum in Mouse Model: Immunoinformatic Standpoint
Source: Biomed Res Int. 2022 Jun 9;2022:2644667. doi: 10.1155/2022/2644667 (PMC9204498; doi:10.1155/2022/2644667)
Supplement: Supplementary 1 — Supplementary File 1. Predicted and screened common linear B-cell epitopes. [file 2644667.f1.docx]

**Table 1.** The most common continuous B-cell epitopes predicted for *N. caninum* **GRA1**, **MIC3**, **MIC6**, **SRS2**, **IMP-1** and **Profilin** proteins, with subsequent screening in terms of antigenicity, allergenicity and water solubility.

| **Examined Protein** | **Shared linear B-cell epitopes** | **VaxiJen antigenicity score** | **AllergenFP allergenicity prediction** | **PepCalc water solubility prediction** |
| --- | --- | --- | --- | --- |
| GRA1 | TSAEEEGTESIPG | 0.9015 | No | Good |
|  | EQHEGDIGYGVR | 1.2830 | No | Good |
|  | DDAAGNPVDSD | 1.4553 | No | Good |
|  | TDGEWPRVVSGQKP | 0.5818 | No | Good |
|  | GLGLARTFRHFV | -1.0073 | Yes | Poor |
|  | DVTDDAITDGE | 1.4481 | Yes | Good |
|  | DIGYGVRAYAGV | 0.3403 | No | Poor |
|  | ALCVCGLAI | 0.2628 | No | Poor |
| MIC3 | DGFMGDGKTCKPDP | 1.4681 | Yes | Good |
|  | GGCGEFCSSSWVFC | 1.5129 | No | Poor |
|  | GYSCKKTGCNAYS | 0.9307 | No | Good |
|  | SEGQPCRNRQLHT | 1.2169 | No | Good |
|  | YTLATDDGTLICA | 0.9818 | No | Poor |
|  | QCHKDAECVED | 1.1507 | Yes | Good |
|  | SCPNGLCCSKT | 0.3984 | No | Poor |
|  | GSNGSCIVVD | 1.4673 | Yes | Poor |
|  | AEALTVQKSS | 0.1214 | Yes | Good |
|  | SQHVEGVAES | 1.4677 | No | Good |
| MIC6 | EPVPERDNTDRTPP | 0.0586 | No | Good |
|  | GFEAVMDATAD | -0.1102 | Yes | Good |
|  | LVNGLASVLSS | 0.0609 | Yes | Poor |
|  | AAGGGAAYM | 0.2612 | No | Poor |
|  | NNACGPANA | 0.3740 | Yes | Poor |
|  | MDATADIKC | 1.3263 | Yes | Good |
|  | GESGEGEE | 3.0951 | No | Good |
|  | TACRSNPC | 0.6338 | Yes | Good |
| SRS2 | ECKERPYSAVFPGF | 1.3682 | No | Good |
|  | FSSSFWTGEASGVA | 1.2775 | No | Poor |
|  | KADAACFAKLSASQ | -0.0722 | No | Good |
|  | VALVYDSQHSIT | 0.6158 | No | Poor |
|  | GPDGKAFPDDY | 1.6063 | No | Good |
|  | VAKPAGAGSN | 1.1244 | Yes | Good |
|  | NNGVTLICGPD | -0.3786 | No | Poor |
|  | VNRSVSVFA | 0.0040 | Yes | Poor |
|  | KAGKNVCLL | 0.4119 | Yes | Good |
| IMP-1 | AVAVAEGLQTAQK | 0.1016 | Yes | Good |
|  | VTEDGDVIVAVDE | 1.0889 | No | Good |
|  | TADSSKGRNSESK | 1.5902 | No | Good |
|  | EKAGKILVSFVPA | 0.6490 | Yes | Good |
|  | MKYEQKGGKTE | 1.5936 | No | Good |
|  | VSPRDLELLRQA | -0.0859 | No | Good |
|  | KSIKGEKTNIV | 1.2111 | No | Good |
|  | STADSSKGRN | 0.9542 | Yes | Good |
| Profilin | QKYKVVRPEKGFEY | -0.2904 | No | Good |
|  | SKLYKEDHEEDT | 0.9530 | No | Good |
|  | CTFDITMCARS | 0.7129 | Yes | Poor |
|  | NEASTIKAAVD | 0.5007 | Yes | Good |
|  | WLVDTGYCCA | 0.8689 | Yes | Poor |
|  | KAAVDDGS | 0.5480 | Yes | Good |
|  | VVKEWLVD | 0.1899 | Yes | Good |
|  | GGIANAEDG | 1.3570 | No | Good |
