# Supplementary material for: Towards the First Multiepitope Vaccine Candidate against Neospora caninum in Mouse Model: Immunoinformatic Standpoint
Source: Biomed Res Int. 2022 Jun 9;2022:2644667. doi: 10.1155/2022/2644667 (PMC9204498; doi:10.1155/2022/2644667)
Supplement: Supplementary 2 — Supplementary File 2. Predicted and screened mouse MHC-I binding epitopes. [file 2644667.f2.docx]

**Table 1.** Prediction of mouse MHC-I-binding epitopes of *N. caninum* **GRA1** using IEDB server followed by antigenicity, allergenicity and toxicity screening

| **Mouse MHC-I alleles** | **Position** | **T-cell peptide** | **Percentile rank** | **VaxiJen antigenicity score** | **AllergenFP allergenicity prediction** | **ToxinPred toxicity prediction** |
| --- | --- | --- | --- | --- | --- | --- |
| **H2-Db** | 39-50 | AVPVVGALTSYL | 3.1 | 0.7984 | No | Non-toxin |
|  | 48-59 | SYLVADRVLPEL | 5.9 | -0.3567 | No | Non-toxin |
|  | 29-40 | AAFAGLGLARTF | 5.4 | -0.7868 | Yes | Non-toxin |
|  | 47-58 | AGVSNYDGDDDA | 7.6 | 0.3022 | No | Non-toxin |
|  | 13-24 | VCGLAIAGLPRL | 7.8 | -0.5198 | Yes | Non-toxin |
| **H2-Dd** | 39-50 | AVPVVGALTSYL | 4.6 | 0.7984 | No | Non-toxin |
|  | 19-30 | AGLPRLASAGDL | 6.1 | -0.4142 | Yes | Non-toxin |
|  | 32-43 | AGLGLARTFRHF | 7.5 | -1.2786 | No | Non-toxin |
|  | 14-25 | SDPEQERAGGPL | 8.1 | 1.2467 | Yes | Non-toxin |
|  | 29-40 | AAFAGLGLARTF | 11 | -0.7868 | Yes | Non-toxin |
| **H2-Kb** | 48-59 | SYLVADRVLPEL | 6.6 | -0.3567 | No | Non-toxin |
|  | 29-40 | AAFAGLGLARTF | 7.5 | -0.7868 | Yes | Non-toxin |
|  | 19-30 | AGLPRLASAGDL | 7.8 | -0.4142 | Yes | Non-toxin |
|  | 23-34 | AALVAAAAFAGL | 8.2 | 0.6714 | No | Non-toxin |
|  | 39-50 | AVPVVGALTSYL | 8.4 | 0.7984 | No | Non-toxin |
| **H2-Kd** | 48-59 | SYLVADRVLPEL | 0.64 | -0.3567 | No | Non-toxin |
|  | 45-56 | ALTSYLVADRVL | 2.9 | -0.0563 | Yes | Non-toxin |
|  | 23-34 | GQKPHTTQKGSL | 5.9 | 1.0132 | Yes | Non-toxin |
|  | 39-50 | AVPVVGALTSYL | 8.8 | 0.7984 | No | Non-toxin |
|  | 29-40 | AAFAGLGLARTF | 14 | -0.7868 | Yes | Non-toxin |
| **H2-Kk** | 15-26 | DPEQERAGGPLI | 2.0 | 0.8222 | Yes | Non-toxin |
|  | 32-43 | TEQHEGDIGYGV | 2.4 | 1.2547 | No | Non-toxin |
|  | 35-46 | HEGDIGYGVRAY | 9.5 | 1.4824 | No | Non-toxin |
|  | 1-12 | NPVDSDVTDDAI | 11 | 0.7513 | No | Non-toxin |
|  | 10-21 | DAITDGEWPRVV | 12 | 1.3603 | Yes | Non-toxin |
| **H2-Ld** | 40-51 | VPVVGALTSYLV | 2.8 | 0.7278 | Yes | Non-toxin |
|  | 39-50 | AVPVVGALTSYL | 3.1 | 0.7984 | No | Non-toxin |
|  | 29-40 | AAFAGLGLARTF | 4.1 | -0.7868 | Yes | Non-toxin |
|  | 48-59 | SYLVADRVLPEL | 7.4 | -0.3567 | No | Non-toxin |
|  | 32-43 | AGLGLARTFRHF | 8.8 | -1.2786 | No | Non-toxin |
| **H2-Qa1** | 29-40 | AAFAGLGLARTF | 3.3 | -0.7868 | Yes | Toxin |
|  | 41-52 | YGVRAYAGVSNY | 4.7 | 0.0660 | No | Non-toxin |
|  | 38-49 | LAVPVVGALTSY | 5.8 | 0.7270 | Yes | Toxin |
|  | 23-34 | GQKPHTTQKGSL | 7.1 | 1.0132 | Yes | Non-toxin |
|  | 47-58 | KSKTVASEDSAL | 8.2 | 1.0215 | Yes | Non-toxin |
| **H2-Qa2** | 32-43 | TEQHEGDIGYGV | 5.2 | 1.2547 | No | Non-toxin |
|  | 35-46 | HEGDIGYGVRAY | 5.8 | 1.0482 | No | Non-toxin |
|  | 23-34 | GQKPHTTQKGSL | 5.9 | 1.0132 | Yes | Non-toxin |
|  | 15-26 | GEWPRVVSGQKP | 6.2 | 0.4746 | Yes | Toxin |
|  | 29-40 | AAFAGLGLARTF | 7.1 | -0.7868 | Yes | Toxin |

**Table 2.** Prediction of mouse MHC-I-binding epitopes of *N. caninum* **MIC3** using IEDB server followed by antigenicity, allergenicity and toxicity screening

| **Mouse MHC-I alleles** | **Position** | **T-cell peptide** | **Percentile rank** | **VaxiJen antigenicity score** | **AllergenFP allergenicity prediction** | **ToxinPred toxicity prediction** |
| --- | --- | --- | --- | --- | --- | --- |
| **H2-Db** | 32-43 | SHLAQLENSQHV | 0.17 | 0.5921 | No | Non-toxin |
|  | 35-46 | AQLENSQHVEGV | 0.27 | 1.1212 | No | Non-toxin |
|  | 88-99 | RNRQLHTNHGYI | 3.2 | 0.6038 | No | Non-toxin |
|  | 65-76 | VSYTLATDDGTL | 3.5 | 0.2033 | No | Non-toxin |
|  | 15-26 | SGAVWMSPAEAL | 5.9 | 0.3771 | Yes | Non-toxin |
| **H2-Dd** | 81-92 | SSEGQPCRNRQL | 2.5 | 1.3015 | Yes | Non-toxin |
|  | 17-28 | AVWMSPAEALTV | 4.7 | 0.3753 | No | Non-toxin |
|  | 32-43 | SHLAQLENSQHV | 7.5 | 0.5921 | No | Non-toxin |
|  | 276-287 | KNPMCYPTCEEM | 8.0 | -0.1100 | No | Toxin |
|  | 65-76 | VSYTLATDDGTL | 8.7 | 0.2033 | No | Non-toxin |
| **H2-Kb** | 65-76 | VSYTLATDDGTL | 2.3 | 0.2033 | No | Non-toxin |
|  | 276-287 | KNPMCYPTCEEM | 7.1 | -0.1100 | No | Toxin |
|  | 15-26 | SGAVWMSPAEAL | 11 | 0.3771 | Yes | Non-toxin |
|  | 26-37 | LTVQKSSHLAQL | 13 | 0.2244 | No | Non-toxin |
|  | 23-34 | AEALTVQKSSHL | 15 | 0.3713 | No | Non-toxin |
| **H2-Kd** | 97-108 | GYITGASCPNGL | 0.49 | 0.8190 | Yes | Non-toxin |
|  | 66-77 | SYTLATDDGTLI | 0.57 | 0.5913 | No | Non-toxin |
|  | 32-43 | SHLAQLENSQHV | 1.6 | 0.5921 | No | Non-toxin |
|  | 241-252 | AYSEYCNPGRCI | 2.2 | 1.1474 | No | Toxin |
|  | 15-26 | SGAVWMSPAEAL | 2.4 | 0.3771 | Yes | Non-toxin |
| **H2-Kk** | 335-346 | AERCEKELGISL | 1.5 | 0.8155 | No | Non-toxin |
|  | 23-34 | AEALTVQKSSHL | 2.0 | 0.3713 | No | Non-toxin |
|  | 323-334 | EEKCVQGAEASL | 2.4 | 1.0084 | No | Non-toxin |
|  | 197-208 | NEKCGSNGSCIV | 6.5 | 1.5425 | No | Non-toxin |
|  | 32-43 | SHLAQLENSQHV | 8.2 | 0.5921 | No | Non-toxin |
| **H2-Ld** | 307-318 | ACDCPAGYSRSM | 2.8 | 0.0745 | No | Toxin |
|  | 15-26 | SGAVWMSPAEAL | 4.2 | 0.3771 | Yes | Non-toxin |
|  | 65-76 | VSYTLATDDGTL | 4.4 | 0.2033 | No | Non-toxin |
|  | 335-346 | AERCEKELGISL | 5.9 | 0.8155 | No | Non-toxin |
|  | 1-12 | MRGGASALVQAL | 6.0 | 0.2523 | Yes | Non-toxin |
| **H2-Qa1** | 45-56 | GVAESLALSPSF | 2.6 | -0.1536 | Yes | Toxin |
|  | 12-23 | STGLIYHADKSY | 4.0 | 0.3074 | No | Non-toxin |
|  | 6-17 | SSWVFCSTGLIY | 4.5 | -0.1274 | Yes | Toxin |
|  | 26-37 | LTVQKSSHLAQL | 7.6 | 0.2244 | No | Non-toxin |
|  | 10-21 | RCIDDASQPSRY | 7.6 | 0.4919 | No | Non-toxin |
| **H2-Qa2** | 23-34 | AEALTVQKSSHL | 2.9 | 0.3713 | No | Non-toxin |
|  | 35-46 | AERCEKELGISL | 3.3 | 0.4855 | No | Non-toxin |
|  | 32-43 | SHLAQLENSQHV | 5.0 | 0.5921 | No | Non-toxin |
|  | 35-46 | AQLENSQHVEGV | 6.8 | 1.1212 | No | Non-toxin |
|  | 47-58 | AESLALSPSFLA | 9.4 | 0.4265 | Yes | Non-toxin |

**Table 3.** Prediction of mouse MHC-I-binding epitopes of *N. caninum* **MIC6** using IEDB server followed by antigenicity, allergenicity and toxicity screening

| **Mouse MHC-I alleles** | **Position** | **T-cell peptide** | **Percentile rank** | **VaxiJen antigenicity score** | **AllergenFP allergenicity prediction** | **ToxinPred toxicity prediction** |
| --- | --- | --- | --- | --- | --- | --- |
| **H2-Db** | 7-18 | AGADNTDDVEVL | 0.68 | 0.4656 | No | Non-toxin |
|  | 25-36 | AAIAGGVIGGLL | 2.4 | 2.1340 | Yes | Non-toxin |
|  | 6-17 | YTPVNGRGGLTC | 2.8 | 0.7606 | No | Non-toxin |
|  | 19-30 | VVSLVNGLASVL | 4.4 | -0.3681 | Yes | Non-toxin |
|  | 9-20 | AAVVAAEGFLWL | 5.2 | 0.3577 | No | Non-toxin |
| **H2-Dd** | 19-30 | RPDTGPGRDRGI | 3.7 | -0.5119 | No | Non-toxin |
|  | 12-23 | RGGLTCKQSRTL | 7.7 | 1.0303 | No | Non-toxin |
|  | 7-18 | AGADNTDDVEVL | 8.4 | 0.4655 | No | Non-toxin |
|  | 18-29 | LWLQNDPRFFVL | 11 | 1.5742 | No | Non-toxin |
|  | 6-17 | YTPVNGRGGLTC | 13 | 0.7606 | No | Non-toxin |
| **H2-Kb** | 18-29 | LWLQNDPRFFVL | 7.4 | 1.5742 | No | Non-toxin |
|  | 27-38 | IAGGVIGGLLLL | 14 | 1.4987 | Yes | Non-toxin |
|  | 9-20 | AAVVAAEGFLWL | 16 | 0.3577 | No | Non-toxin |
|  | 5-16 | QGYDLGVENDQV | 19 | 0.2266 | No | Non-toxin |
|  | 15-26 | EGFLWLQNDPRF | 21 | 0.7473 | No | Non-toxin |
| **H2-Kd** | 25-36 | RFFVLPGNGQVA | 9.3 | 0.3127 | Yes | Non-toxin |
|  | 18-29 | LWLQNDPRFFVL | 9.4 | 1.5742 | No | Non-toxin |
|  | 17-28 | FLWLQNDPRFFV | 14 | 1.4389 | No | Non-toxin |
|  | 11-22 | VENDQVTCVVSL | 18 | 0.6346 | No | Non-toxin |
|  | 25-36 | AAIAGGVIGGLL | 18 | 2.1340 | Yes | Non-toxin |
| **H2-Kk** | 11-22 | VENDQVTCVVSL | 1.2 | 0.6346 | No | Non-toxin |
|  | 2-14 | DLGVENDQVTCV | 3.7 | 0.8528 | No | Non-toxin |
|  | 4-16 | GEEDGSREDEPV | 6.5 | 1.4625 | Yes | Non-toxin |
|  | 6-15 | VEVLVDMDSKTW | 6.6 | 1.3409 | No | Non-toxin |
|  | 3-28 | CENNACGPANAV | 7.5 | 1.0298 | Yes | Non-toxin |
| **H2-Ld** | 42-53 | CRPVGYSGRVCL | 4.3 | 1.4002 | No | Non-toxin |
|  | 19-30 | RPDTGPGRDRGI | 4.9 | -0.5119 | No | Non-toxin |
|  | 43-54 | RPVGYSGRVCLC | 6.2 | 1.1177 | Yes | Non-toxin |
|  | 42-53 | KPCGPDEAGSCV | 9.3 | 0.7293 | Yes | Non-toxin |
|  | 18-29 | LWLQNDPRFFVL | 11 | 1.5742 | No | Non-toxin |
| **H2-Qa1** | 8-19 | VAAVVAAEGFLW | 7.3 | 0.4757 | No | Non-toxin |
|  | 36-47 | LLLGAAGGGAAY | 7.9 | 0.6974 | No | Non-toxin |
|  | 26-37 | AIAGGVIGGLLL | 8.7 | 1.9985 | Yes | Non-toxin |
|  | 27-38 | IAGGVIGGLLLL | 9.1 | 1.4987 | Yes | Non-toxin |
|  | 15-26 | VEVLVDMDSKTW | 11 | 1.3409 | Yes | Non-toxin |
| **H2-Qa2** | 11-22 | VENDQVTCVVSL | 1.9 | 0.6346 | No | Non-toxin |
|  | 15-26 | VEVLVDMDSKTW | 2.1 | 1.3409 | Yes | Non-toxin |
|  | 40-51 | GEEDGSREDEPV | 5.7 | 1.4625 | Yes | Non-toxin |
|  | 46-57 | REDEPVPERDNT | 7.9 | 0.4022 | Yes | Non-toxin |
|  | 48-59 | AGVCEDATSGYI | 9.0 | 1.2368 | No | Non-toxin |

**Table 4.** Prediction of mouse MHC-I-binding epitopes of *N. caninum* **SRS2** using IEDB server followed by antigenicity, allergenicity and toxicity screening

| **Mouse MHC-I alleles** | **Position** | **T-cell peptide** | **Percentile rank** | **VaxiJen antigenicity score** | **AllergenFP allergenicity prediction** | **ToxinPred toxicity prediction** |
| --- | --- | --- | --- | --- | --- | --- |
| **H2-Db** | 24-35 | ITVNPENNGVTL | 0.61 | 1.3534 | No | Non-toxin |
|  | 44-55 | VGCKAGKNVCLL | 5.4 | 0.2220 | Yes | Non-toxin |
|  | 4-15 | YSAVFPGFSSSF | 5.4 | 0.4492 | No | Non-toxin |
|  | 47-58 | KAGKNVCLLNVY | 6.7 | -0.0962 | No | Non-toxin |
|  | 10-21 | VAHCAYSSNVRL | 7.7 | 1.5329 | No | Non-toxin |
| **H2-Dd** | 4-15 | YSAVFPGFSSSF | 0.58 | 0.4492 | No | Non-toxin |
|  | 6-17 | LSTFLPGAKKEW | 4.7 | 0.0692 | Yes | Non-toxin |
|  | 48-59 | GHPDDKQVTCVV | 4.7 | 1.7670 | No | Non-toxin |
|  | 24-35 | ITVNPENNGVTL | 5.5 | 1.3534 | No | Non-toxin |
|  | 26-37 | VNPENNGVTLIC | 6.7 | 1.0295 | No | Non-toxin |
| **H2-Kb** | 10-21 | VAHCAYSSNVRL | 2.7 | 1.5329 | No | Non-toxin |
|  | 31-42 | SVNRSVSVFALL | 4.0 | -0.3061 | No | Non-toxin |
|  | 4-15 | YSAVFPGFSSSF | 5.1 | 0.4492 | No | Non-toxin |
|  | 6-17 | AVFPGFSSSFWT | 7.9 | 0.8884 | No | Non-toxin |
|  | 32-43 | VNRSVSVFALLF | 9.3 | 0.1617 | No | Non-toxin |
| **H2-Kd** | 24-35 | SYVALSAASLTA | 2.9 | 0.4534 | No | Non-toxin |
|  | 4-15 | YSAVFPGFSSSF | 6.0 | 0.4492 | No | Non-toxin |
|  | 33-44 | EHYPATSKAFRV | 6.1 | 0.3313 | No | Non-toxin |
|  | 33-44 | DQFPSTAQTIYL | 6.6 | -0.0877 | No | Non-toxin |
|  | 31-42 | PKDQFPSTAQTI | 7.0 | 0.1665 | No | Non-toxin |
| **H2-Kk** | 25-36 | TVNPENNGVTLI | 1.4 | 0.9366 | No | Non-toxin |
|  | 17-28 | DACDELPSYVAL | 1.6 | 0.1381 | Yes | Non-toxin |
|  | 3-14 | ETGGENGDSPVL | 3.1 | 1.1581 | Yes | Non-toxin |
|  | 33-44 | DQFPSTAQTIYL | 3.9 | -0.0877 | No | Non-toxin |
|  | 15-26 | KEWVTGTLQQGI | 4.9 | 1.2743 | No | Non-toxin |
| **H2-Ld** | 36-47 | LPSKLLSEDDGL | 0.42 | 0.0641 | Yes | Non-toxin |
|  | 22-33 | LPSYVALSAASL | 0.92 | 0.2773 | No | Non-toxin |
|  | 24-35 | ITVNPENNGVTL | 1.2 | 1.3534 | No | Non-toxin |
|  | 11-22 | SPVLRGDACDEL | 1.3 | 1.3596 | No | Non-toxin |
|  | 22-33 | RPITVNPENNGV | 1.7 | 1.3057 | Yes | Non-toxin |
| **H2-Qa1** | 4-15 | YSAVFPGFSSSF | 2.1 | 0.4492 | No | Non-toxin |
|  | 17-28 | ALVYDSQHSITF | 3.3 | 0.4102 | No | Non-toxin |
|  | 33-44 | VTLICGPDGKAF | 5.1 | 0.4982 | Yes | Non-toxin |
|  | 24-35 | ITVNPENNGVTL | 5.4 | 1.3543 | No | Non-toxin |
|  | 5-16 | SAVFPGFSSSFW | 5.4 | 1.0065 | No | Non-toxin |
| **H2-Qa2** | 25-36 | TVNPENNGVTLI | 2.5 | 0.9366 | No | Non-toxin |
|  | 15-26 | KEWVTGTLQQGI | 3.1 | 1.2743 | No | Non-toxin |
|  | 17-28 | ALVYDSQHSITF | 3.1 | 0.4102 | No | Non-toxin |
|  | 33-44 | DACDELPSYVAL | 4.1 | 0.1381 | Yes | Non-toxin |
|  | 3-14 | DQFPSTAQTIYL | 5.3 | -0.0877 | No | Non-toxin |

**Table 5.** Prediction of mouse MHC-I-binding epitopes of *N. caninum* **Immune Mapped Protein-1 (IMP-1)** using IEDB server followed by antigenicity, allergenicity and toxicity screening

| **Mouse MHC-I alleles** | **Position** | **T-cell peptide** | **Percentile rank** | **VaxiJen antigenicity score** | **AllergenFP allergenicity prediction** | **ToxinPred toxicity prediction** |
| --- | --- | --- | --- | --- | --- | --- |
| **H2-Db** | 12-23 | VEHVSPRDLELL | 2.9 | 0.1894 | Yes | Non-toxin |
|  | 24-35 | VVSRSPEPAAVI | 4.8 | 0.2167 | No | Non-toxin |
|  | 4-15 | YVPDKGGSLILL | 5.6 | -0.8740 | No | Non-toxin |
|  | 46-57 | GAPAAVTEDGDV | 5.8 | 1.2707 | Yes | Non-toxin |
|  | 19-30 | VAVVPADKSKEF | 6.8 | 0.4950 | No | Non-toxin |
| **H2-Dd** | 4-15 | YVPDKGGSLILL | 1.1 | -0.8740 | No | Non-toxin |
|  | 12-23 | VEHVSPRDLELL | 1.5 | 0.1894 | Yes | Non-toxin |
|  | 24-35 | VVSRSPEPAAVI | 2.0 | 0.2167 | No | Non-toxin |
|  | 41-52 | TEDMPPQVFLSL | 2.6 | 1.0264 | Yes | Non-toxin |
|  | 1-12 | YLLYVPDKGGSL | 2.9 | -0.0065 | No | Non-toxin |
| **H2-Kb** | 4-15 | YVPDKGGSLILL | 6.2 | -0.8740 | No | Non-toxin |
|  | 29-40 | AGKILVSFVPAL | 8.2 | 0.6598 | Yes | Non-toxin |
|  | 11-22 | VDLSVFSHVAVV | 9.1 | 0.6512 | No | Non-toxin |
|  | 36-47 | FVPALHKSVPRM | 11 | -0.1124 | Yes | Non-toxin |
|  | 41-52 | TEDMPPQVFLSL | 11 | 1.0264 | Yes | Non-toxin |
| **H2-Kd** | 14-25 | KQKYYAAWATVL | 1.4 | 0.4647 | Yes | Non-toxin |
|  | 48-59 | KYEQKGGKTELL | 1.9 | 0.9165 | Yes | Non-toxin |
|  | 3-14 | LYVPDKGGSLIL | 2.0 | -0.6096 | No | Non-toxin |
|  | 39-50 | NWTEDMPPQVFL | 3.4 | 1.7663 | Yes | Non-toxin |
|  | 2-13 | LLYVPDKGGSLI | 7.6 | -0.4493 | Yes | Non-toxin |
| **H2-Kk** | 41-52 | TEDMPPQVFLSL | 0.87 | 1.0264 | Yes | Non-toxin |
|  | 20-31 | SKATEAERAVAV | 1.6 | 1.0536 | Yes | Non-toxin |
|  | 18-29 | SESKATEAERAV | 2.0 | 1.0414 | Yes | Non-toxin |
|  | 25-36 | EEEKAGKILVSF | 2.2 | 1.1575 | No | Non-toxin |
|  | 29-40 | DEYEATLCVRNW | 2.2 | 1.1189 | No | Non-toxin |
| **H2-Ld** | 41-52 | TEDMPPQVFLSL | 0.61 | 1.0264 | Yes | Non-toxin |
|  | 44-55 | MPPQVFLSLLHV | 1.1 | 0.6408 | No | Non-toxin |
|  | 5-16 | LPRDRPVDLSVF | 1.3 | 0.9249 | No | Non-toxin |
|  | 5-16 | VPDKGGSLILLW | 1.5 | -0.9925 | Yes | Non-toxin |
|  | 19-30 | VAVVPADKSKEF | 1.5 | 0.4950 | No | Non-toxin |
| **H2-Qa1** | 4-15 | YVPDKGGSLILL | 3.0 | -0.8740 | No | Non-toxin |
|  | 7-18 | SVWKANEKQKYY | 3.9 | 0.2189 | No | Non-toxin |
|  | 41-52 | VTNSDGQNRREY | 4.1 | 0.3667 | No | Non-toxin |
|  | 1-12 | YLLYVPDKGGSL | 5.7 | -0.0065 | No | Non-toxin |
|  | 10-21 | KANEKQKYYAAW | 5.9 | 0.5640 | No | Non-toxin |
| **H2-Qa2** | 41-52 | TEDMPPQVFLSL | 1.1 | 1.0264 | Yes | Non-toxin |
|  | 38-49 | RNWTEDMPPQVF | 1.3 | 1.2711 | No | Non-toxin |
|  | 27-38 | REQVKATGGPVI | 1.5 | 0.9087 | Yes | Non-toxin |
|  | 25-36 | EEEKAGKILVSF | 2.2 | 1.1575 | No | Non-toxin |
|  | 29-40 | DEYEATLCVRNW | 2.8 | 1.1189 | No | Non-toxin |

**Table 6.** Prediction of mouse MHC-I-binding epitopes of *N. caninum* **Profilin** using IEDB server followed by antigenicity, allergenicity and toxicity screening

| **Mouse MHC-I alleles** | **Position** | **T-cell peptide** | **Percentile rank** | **VaxiJen antigenicity score** | **AllergenFP allergenicity prediction** | **ToxinPred toxicity prediction** |
| --- | --- | --- | --- | --- | --- | --- |
| **H2-Db** | 27-38 | SRTSALAFAEYL | 1.2 | 0.8596 | No | Non-toxin |
|  | 17-28 | VDDGSAPNGVWI | 1.3 | 1.1841 | No | Non-toxin |
|  | 21-32 | GGIANAEDGVVF | 1.4 | 0.7984 | No | Non-toxin |
|  | 1-12 | AHLIKTPNGSIV | 5.0 | 0.2470 | No | Non-toxin |
|  | 1-12 | MSDWDPVVKEWL | 6.5 | 0.2082 | No | Non-toxin |
| **H2-Dd** | 1-12 | MSDWDPVVKEWL | 6.3 | 0.2082 | No | Non-toxin |
|  | 5-16 | KTPNGSIVIALY | 7.1 | 1.0959 | Yes | Non-toxin |
|  | 2-13 | HLIKTPNGSIVI | 8.6 | 0.0488 | No | Non-toxin |
|  | 16-27 | AVDDGSAPNGVW | 9.2 | 0.6596 | No | Non-toxin |
|  | 4-15 | IKTPNGSIVIAL | 11 | 0.4915 | Yes | Non-toxin |
| **H2-Kb** | 27-38 | SRTSALAFAEYL | 8.8 | 0.8596 | No | Non-toxin |
|  | 5-16 | KTPNGSIVIALY | 11 | 1.0959 | Yes | Non-toxin |
|  | 34-45 | AAADDDDGWSKL | 14 | -0.0593 | No | Non-toxin |
|  | 4-15 | IKTPNGSIVIAL | 19 | 0.4915 | Yes | Non-toxin |
|  | 42-35 | FEYNDCTFDITM | 20 | 0.2551 | No | Non-toxin |
| **H2-Kd** | 1-12 | AHLIKTPNGSIV | 2.9 | 0.2470 | No | Non-toxin |
|  | 27-38 | SRTSALAFAEYL | 8.2 | 0.8596 | No | Non-toxin |
|  | 40-51 | KGFEYNDCTFDI | 11 | 0.2441 | No | Non-toxin |
|  | 44-55 | KLYKEDHEEDTI | 11 | 0.7124 | No | Non-toxin |
|  | 2-13 | HLIKTPNGSIVI | 14 | 0.0488 | No | Non-toxin |
| **H2-Kk** | 42-53 | FEYNDCTFDITM | 3.4 | 0.2551 | No | Non-toxin |
|  | 2-13 | SDWDPVVKEWLV | 5.5 | 0.2873 | Yes | Non-toxin |
|  | 21-32 | EQDKGNSRTSAL | 6.9 | 0.9240 | Yes | Non-toxin |
|  | 22-33 | APNGVWIGGQKY | 11 | 0.8319 | Yes | Non-toxin |
|  | 34-45 | AAADDDDGWSKL | 11 | -0.0593 | No | Non-toxin |
| **H2-Ld** | 22-33 | APNGVWIGGQKY | 2.9 | 0.8319 | Yes | Non-toxin |
|  | 2-13 | SDWDPVVKEWLV | 7.3 | 0.2873 | Yes | Non-toxin |
|  | 4-15 | IKTPNGSIVIAL | 8.0 | 0.4915 | Yes | Non-toxin |
|  | 27-38 | SRTSALAFAEYL | 8.0 | 0.8596 | No | Non-toxin |
|  | 1-12 | MSDWDPVVKEWL | 12 | 0.2082 | No | Non-toxin |
| **H2-Qa1** | 5-16 | KTPNGSIVIALY | 2.8 | 1.0959 | Yes | Non-toxin |
|  | 16-27 | AVDDGSAPNGVW | 6.1 | 0.6569 | No | Non-toxin |
|  | 33-44 | YKVVRPEKGFEY | 6.4 | -0.8100 | Yes | Non-toxin |
|  | 22-33 | APNGVWIGGQKY | 6.6 | 0.8319 | No | Non-toxin |
|  | 35-46 | AADDDDGWSKLY | 11 | -0.3343 | No | Non-toxin |
| **H2-Qa2** | 2-13 | SDWDPVVKEWLV | 3.1 | 0.2873 | Yes | Non-toxin |
|  | 42-53 | FEYNDCTFDITM | 4.9 | 0.2551 | No | Non-toxin |
|  | 20-31 | KEQDKGNSRTSA | 8.6 | 1.2858 | No | Non-toxin |
|  | 21-32 | EQDKGNSRTSAL | 9.0 | 0.9240 | Yes | Non-toxin |
|  | 34-45 | AAADDDDGWSKL | 9.7 | -0.0593 | No | Non-toxin |
