# Supplementary material for: Towards the First Multiepitope Vaccine Candidate against Neospora caninum in Mouse Model: Immunoinformatic Standpoint
Source: Biomed Res Int. 2022 Jun 9;2022:2644667. doi: 10.1155/2022/2644667 (PMC9204498; doi:10.1155/2022/2644667)
Supplement: Supplementary 3 — Supplementary File 3. Predicted and screened mouse MHC-II binding epitopes. [file 2644667.f3.docx]

**Table 1.** Prediction of mouse MHC-II-binding epitopes of *N. caninum* **GRA1** using IEDB server followed by screening for antigenicity, allergenicity and IFN-γ induction

| **Mouse MHC-II alleles** | **Position** | **T-cell peptide** | **Percentile rank** | **VaxiJen antigenicity score** | **AllergenFP allergenicity prediction** | **ToxinPred toxicity prediction** |
| --- | --- | --- | --- | --- | --- | --- |
| **H2-IAb** | 37-51 | ARTFRHFVPKKSKTV | 4.9 | 0.3055 | No | Non-toxin |
|  | 19-33 | AVGIAALVAAAAFAG | 5.1 | 0.5883 | No | Non-toxin |
|  | 38-52 | RTFRHFVPKKSKTVA | 5.3 | 0.3271 | No | Non-toxin |
|  | 20-34 | VGIAALVAAAAFAGL | 5.5 | 0.3041 | No | Non-toxin |
|  | 14-28 | CGLAIAGLPRLASAG | 6.1 | -0.0641 | No | Non-toxin |
|  | 16-30 | VKTAVGIAALVAAAA | 6.3 | 0.3051 | No | Non-toxin |
| **H2-IAd** | 16-30 | VKTAVGIAALVAAAA | 1.5 | 0.3051 | No | Non-toxin |
|  | 17-31 | KTAVGIAALVAAAAF | 1.65 | 0.4107 | No | Non-toxin |
|  | 18-32 | TAVGIAALVAAAAFA | 2.25 | 0.4706 | No | Non-toxin |
|  | 19-33 | AVGIAALVAAAAFAG | 2.3 | 0.5883 | No | Non-toxin |
|  | 13-27 | KKRVKTAVGIAALVA | 2.55 | 0.8230 | No | Non-toxin |
|  | 14-28 | KRVKTAVGIAALVAA | 2.7 | 0.5602 | No | Non-toxin |
| **H2-IEd** | 38-52 | RTFRHFVPKKSKTVA | 1.2 | 0.3271 | No | Non-toxin |
|  | 37-51 | ARTFRHFVPKKSKTV | 1.21 | 0.3055 | No | Non-toxin |
|  | 36-50 | LARTFRHFVPKKSKT | 1.6 | 0.0782 | No | Non-toxin |
|  | 39-53 | TFRHFVPKKSKTVAS | 1.75 | 0.5940 | No | Non-toxin |
|  | 35-49 | GLARTFRHFVPKKSK | 2.25 | -0.4796 | Yes | Non-toxin |
|  | 34-48 | LGLARTFRHFVPKKS | 2.3 | -0.8126 | No | Non-toxin |

**Table 2.** Prediction of mouse MHC-II-binding epitopes of *N. caninum* **MIC3** using IEDB server followed by screening for antigenicity, allergenicity and IFN-γ induction

| **Mouse MHC-II alleles** | **Position** | **T-cell peptide** | **Percentile rank** | **VaxiJen antigenicity score** | **AllergenFP allergenicity prediction** | **ToxinPred toxicity prediction** |
| --- | --- | --- | --- | --- | --- | --- |
| **H2-IAb** | 14-28 | VSGAVWMSPAEALTV | 2.00 | 0.5968 | No | Non-toxin |
|  | 15-29 | SGAVWMSPAEALTVQ | 2.20 | 0.3891 | Yes | Non-toxin |
|  | 13-27 | TVSGAVWMSPAEALT | 2.75 | 0.6804 | No | Non-toxin |
|  | 12-26 | LTVSGAVWMSPAEAL | 3.00 | 0.8022 | No | Non-toxin |
|  | 52-66 | LSPSFLASGISSEVS | 3.3 | 1.0545 | No | Non-toxin |
|  | 51-65 | ALSPSFLASGISSEV | 3.45 | 1.1256 | No | Non-toxin |
| **H2-IAd** | 17-31 | AVWMSPAEALTVQKS | 3.45 | 0.1773 | No | Non-toxin |
|  | 3-17 | GGASALVQALTVSGA | 3.75 | 0.4609 | No | Non-toxin |
|  | 4-18 | GASALVQALTVSGAV | 3.94 | 0.6161 | No | Non-toxin |
|  | 18-32 | VWMSPAEALTVQKSS | 4.24 | 0.2266 | No | Non-toxin |
|  | 5-19 | ASALVQALTVSGAVW | 4.3 | 0.8968 | No | Non-toxin |
|  | 15-29 | SGAVWMSPAEALTVQ | 4.48 | 0.3891 | Yes | Non-toxin |
| **H2-IEd** | 263-277 | CECPQDSWRTTEGKN | 18 | 0.7478 | Yes | Non-toxin |
|  | 265-279 | CPQDSWRTTEGKNPM | 20 | 0.2257 | No | Non-toxin |
|  | 264-278 | ECPQDSWRTTEGKNP | 21 | 0.6233 | No | Non-toxin |
|  | 167-181 | NAGGSVRCRCKDGFM | 23.25 | 1.1988 | No | Non-toxin |
|  | 168-182 | AGGSVRCRCKDGFMG | 24 | 1.7332 | No | Non-toxin |
|  | 262-276 | TCECPQDSWRTTEGK | 24 | 0.8508 | No | Non-toxin |

**Table 3.** Prediction of mouse MHC-II-binding epitopes of *N. caninum* **MIC6** using IEDB server followed by screening for antigenicity, allergenicity and IFN-γ induction

| **Mouse MHC-II alleles** | **Position** | **T-cell peptide** | **Percentile rank** | **VaxiJen antigenicity score** | **AllergenFP allergenicity prediction** | **ToxinPred toxicity prediction** |
| --- | --- | --- | --- | --- | --- | --- |
| **H2-IAb** | 15-29 | GKKEESKGSAAAIAG | 6.5 | 1.5561 | No | Non-toxin |
|  | 16-30 | KKEESKGSAAAIAGG | 7.45 | 1.4668 | No | Non-toxin |
|  | 14-28 | GGKKEESKGSAAAIA | 7.75 | 1.3135 | Yes | Non-toxin |
|  | 17-31 | KEESKGSAAAIAGGV | 8.15 | 1.4473 | No | Non-toxin |
|  | 35-49 | LLLLGAAGGGAAYMM | 8.35 | 0.4788 | Yes | Non-toxin |
|  | 24-38 | NGLASVLSSSKTTAC | 8.6 | 0.2380 | No | Non-toxin |
| **H2-IAd** | 2-16 | WLFRNCVAAVVAAEG | 7.15 | 0.5728 | No | Non-toxin |
|  | 1-15 | MWLFRNCVAAVVAAE | 7.21 | 0.6471 | No | Non-toxin |
|  | 3-17 | LFRNCVAAVVAAEGF | 8.15 | 0.3784 | No | Non-toxin |
|  | 17-31 | TCVVSLVNGLASVLS | 10.35 | -0.3107 | No | Non-toxin |
|  | 4-18 | FRNCVAAVVAAEGFL | 12.75 | 0.7991 | No | Non-toxin |
|  | 13-27 | NDQVTCVVSLVNGLA | 13.5 | 0.1996 | No | Non-toxin |
| **H2-IEd** | 13-27 | AAEGFLWLQNDPRFF | 13.25 | 0.5652 | No | Non-toxin |
|  | 14-28 | AEGFLWLQNDPRFFV | 13.56 | 0.9106 | No | Non-toxin |
|  | 42-56 | GGGAAYMMKSKGNDE | 14.75 | 0.3525 | No | Non-toxin |
|  | 43-57 | GGAAYMMKSKGNDES | 16.5 | 0.5248 | No | Non-toxin |
|  | 7-21 | DIKCKPASPHRHRPD | 17.1 | 0.7295 | No | Non-toxin |
|  | 8-22 | IKCKPASPHRHRPDT | 17.15 | 0.9236 | Yes | Non-toxin |

**Table 4.** Prediction of mouse MHC-II-binding epitopes of *N. caninum* **SRS2** using IEDB server followed by screening for antigenicity, allergenicity and IFN-γ induction

| **Mouse MHC-II alleles** | **Position** | **T-cell peptide** | **Percentile rank** | **VaxiJen antigenicity score** | **AllergenFP allergenicity prediction** | **ToxinPred toxicity prediction** |
| --- | --- | --- | --- | --- | --- | --- |
| **H2-IAb** | 23-37 | PSYVALSAASLTATA | 1.1 | 0.4796 | No | Non-toxin |
|  | 22-36 | LPSYVALSAASLTAT | 1.21 | 0.3898 | No | Non-toxin |
|  | 24-38 | SYVALSAASLTATAI | 1.41 | 0.4271 | No | Non-toxin |
|  | 21-35 | ELPSYVALSAASLTA | 1.5 | 0.4580 | No | Non-toxin |
|  | 25-39 | YVALSAASLTATAIF | 1.76 | 0.3833 | No | Non-toxin |
|  | 2-34 | DELPSYVALSAASLT | 1.85 | 0.2164 | No | Non-toxin |
| **H2-IAd** | 20-34 | DELPSYVALSAASLT | 0.64 | 0.2164 | No | Non-toxin |
|  | 21-35 | ELPSYVALSAASLTA | 0.73 | 0.4580 | No | Non-toxin |
|  | 22-36 | LPSYVALSAASLTAT | 0.73 | 0.3898 | No | Non-toxin |
|  | 23-37 | PSYVALSAASLTATA | 0.98 | 0.4796 | No | Non-toxin |
|  | 24-38 | SYVALSAASLTATAI | 1.39 | 0.4271 | No | Non-toxin |
|  | 19-33 | CDELPSYVALSAASL | 1.59 | 0.3479 | No | Non-toxin |
| **H2-IEd** | 1-15 | MATHACVVRRKADAA | 1.99 | -0.3581 | No | Non-toxin |
|  | 2-16 | ATHACVVRRKADAAC | 2.75 | -0.5192 | Yes | Non-toxin |
|  | 12-26 | HCAYSSNVRLRPITV | 3.00 | 1.3421 | No | Non-toxin |
|  | 11-25 | AHCAYSSNVRLRPIT | 3.35 | 1.4654 | No | Non-toxin |
|  | 3-17 | THACVVRRKADAACF | 3.9 | -0.6533 | Yes | Non-toxin |
|  | 10-24 | VAHCAYSSNVRLRPI | 4.1 | 1.3060 | No | Non-toxin |

**Table 5.** Prediction of mouse MHC-II-binding epitopes of *N. caninum* **Immune Mapped Protein-1 (IMP-1)** using IEDB server followed by screening for antigenicity, allergenicity and IFN-γ induction

| **Mouse MHC-II alleles** | **Position** | **T-cell peptide** | **Percentile rank** | **VaxiJen antigenicity score** | **AllergenFP allergenicity prediction** | **ToxinPred toxicity prediction** |
| --- | --- | --- | --- | --- | --- | --- |
| **H2-IAb** | 13-27 | EKQKYYAAWATVLKT | 0.5 | 0.0589 | No | Non-toxin |
|  | 12-26 | NEKQKYYAAWATVLK | 0.58 | 0.1720 | Yes | Non-toxin |
|  | 11-25 | ANEKQKYYAAWATVL | 0.72 | 0.4536 | No | Non-toxin |
|  | 14-28 | KQKYYAAWATVLKTC | 0.8 | 0.1605 | No | Non-toxin |
|  | 15-29 | QKYYAAWATVLKTCD | 0.91 | -0.0727 | Yes | Non-toxin |
|  | 39-53 | QKIEAVTGAPAAVTE | 0.94 | 0.3219 | No | Non-toxin |
| **H2-IAd** | 13-27 | EKQKYYAAWATVLKT | 1.62 | 0.0589 | No | Non-toxin |
|  | 14-28 | KQKYYAAWATVLKTC | 1.75 | 0.1605 | No | Non-toxin |
|  | 15-29 | QKYYAAWATVLKTCD | 2.10 | -0.0727 | Yes | Non-toxin |
|  | 12-26 | NEKQKYYAAWATVLK | 2.18 | 0.1720 | Yes | Non-toxin |
|  | 13-27 | LSVFSHVAVVPADKS | 2.77 | 0.4540 | No | Non-toxin |
|  | 12-26 | DLSVFSHVAVVPADK | 3.2 | 0.5379 | No | Non-toxin |
| **H2-IEd** | 35-49 | SFVPALHKSVPRMKY | 1.5 | 0.0715 | No | Non-toxin |
|  | 34-48 | VSFVPALHKSVPRMK | 2.05 | 0.0013 | No | Non-toxin |
|  | 36-50 | FVPALHKSVPRMKYE | 2.35 | 0.1416 | No | Non-toxin |
|  | 2-16 | IEDKWSVWKANEKQK | 2.6 | 0.2682 | No | Non-toxin |
|  | 13-27 | EKQKYYAAWATVLKT | 2.75 | 0.0589 | No | Non-toxin |
|  | 14-28 | KQKYYAAWATVLKTC | 2.9 | 0.1605 | No | Non-toxin |

**Table 6.** Prediction of mouse MHC-II-binding epitopes of *N. caninum* **Profilin** using IEDB server followed by screening for antigenicity, allergenicity and IFN-γ induction

| **Mouse MHC-II alleles** | **Position** | **T-cell peptide** | **Percentile rank** | **VaxiJen antigenicity score** | **AllergenFP allergenicity prediction** | **ToxinPred toxicity prediction** |
| --- | --- | --- | --- | --- | --- | --- |
| **H2-IAb** | 1-15 | AHLIKTPNGSIVIAL | 6.8 | 0.3584 | No | Non-toxin |
|  | 2-16 | HLIKTPNGSIVIALY | 7.25 | 0.4429 | Yes | Non-toxin |
|  | 3-17 | GKVTVNEASTIKAAV | 9.4 | 0.5204 | No | Non-toxin |
|  | 3-17 | LIKTPNGSIVIALYD | 9.85 | 0.4190 | Yes | Non-toxin |
|  | 2-16 | NGKVTVNEASTIKAA | 10.25 | 0.6971 | No | Non-toxin |
|  | 4-18 | KVTVNEASTIKAAVD | 12.00 | 0.4181 | Yes | Non-toxin |
| **H2-IAd** | 4-18 | KVTVNEASTIKAAVD | 5.45 | 0.4181 | Yes | Non-toxin |
|  | 5-19 | VTVNEASTIKAAVDD | 5.45 | 0.4044 | No | Non-toxin |
|  | 6-20 | TVNEASTIKAAVDDG | 7.25 | 0.3376 | No | Non-toxin |
|  | 3-17 | GKVTVNEASTIKAAV | 8.35 | 0.5204 | No | Non-toxin |
|  | 2-16 | NGKVTVNEASTIKAA | 10.9 | 0.6971 | No | Non-toxin |
|  | 7-21 | VNEASTIKAAVDDGS | 11 | 0.4311 | No | Non-toxin |
| **H2-IEd** | 28-42 | IGGQKYKVVRPEKGF | 3.35 | 0.0865 | No | Non-toxin |
|  | 29-43 | GGQKYKVVRPEKGFE | 3.8 | -0.2746 | No | Non-toxin |
|  | 27-41 | WIGGQKYKVVRPEKG | 4.65 | 0.1479 | Yes | Non-toxin |
|  | 30-44 | GQKYKVVRPEKGFEY | 5.35 | 0.4179 | No | Non-toxin |
|  | 26-40 | VWIGGQKYKVVRPEK | 6.05 | 0.8415 | No | Non-toxin |
|  | 25-39 | GVWIGGQKYKVVRPE | 9.75 | 0.9887 | No | Non-toxin |
