# Supplementary material for: Towards the First Multiepitope Vaccine Candidate against Neospora caninum in Mouse Model: Immunoinformatic Standpoint
Source: Biomed Res Int. 2022 Jun 9;2022:2644667. doi: 10.1155/2022/2644667 (PMC9204498; doi:10.1155/2022/2644667)
Supplement: Supplementary 4 — Supplementary File 4. Predicted and screened IFN-γ-inducing epitopes. [file 2644667.f4.docx]

| No. | Epitope | Score | Antigenicity | Allergenicity | Toxicity |
| --- | --- | --- | --- | --- | --- |
| 1 | [FPRAVRRAVSVGVFA](http://crdd.osdd.net/raghava/ifnepitope/pep_design.php?sequence=FPRAVRRAVSVGVFA&method=hybrid&model=main) | 1.0249248 | 0.5431 | No | Non-Toxin |
| 2 | [PRAVRRAVSVGVFAA](http://crdd.osdd.net/raghava/ifnepitope/pep_design.php?sequence=PRAVRRAVSVGVFAA&method=hybrid&model=main) | \|  \| 1.1840912 \| \| --- \| --- \| | 0.5884 | No | Non-Toxin |
| 3 | [RAVRRAVSVGVFAAP](http://crdd.osdd.net/raghava/ifnepitope/pep_design.php?sequence=RAVRRAVSVGVFAAP&method=hybrid&model=main) | 1.1697089 | 0.7572 | No | Non-Toxin |
| 4 | [SLFAVTIGLVGSIAA](http://crdd.osdd.net/raghava/ifnepitope/pep_design.php?sequence=SLFAVTIGLVGSIAA&method=hybrid&model=main) | 0.1062379 | 0.6322 | No | Non-Toxin |
| 5 | \|  \| [KSSAENVGRVSLFAV](http://crdd.osdd.net/raghava/ifnepitope/pep_design.php?sequence=KSSAENVGRVSLFAV&method=hybrid&model=main) \| \| --- \| --- \| | \|  \| 0.16519958 \| \| --- \| --- \| | 0.0540 | No | Non-Toxin |
| 6 | [SAGIKSSAENVGRVS](http://crdd.osdd.net/raghava/ifnepitope/pep_design.php?sequence=SAGIKSSAENVGRVS&method=hybrid&model=main) | 0.19257684 | 0.6960 | No | Non-Toxin |
| 7 | [ASAGIKSSAENVGRV](http://crdd.osdd.net/raghava/ifnepitope/pep_design.php?sequence=ASAGIKSSAENVGRV&method=hybrid&model=main) | 0.31839436 | 0.6057 | No | Non-Toxin |
| 8 | [EAERASAGIKSSAEN](http://crdd.osdd.net/raghava/ifnepitope/pep_design.php?sequence=EAERASAGIKSSAEN&method=hybrid&model=main) | 0.42062559 | 0.9542 | No | Non-Toxin |
